# The role of public and patient involvement in designing a web-based, physical activity application for individuals with severe mental illness

**DOI:** 10.1186/s40900-025-00735-x

**Published:** 2025-07-21

**Authors:** Shannon Aisling Forde, Susan Rea, Emmy Racine, Tara Coppinger

**Affiliations:** 1 Department of Sport, Leisure & Childhood Studies, Munster Technological University, Bishopstown, Cork, T12 P928 Ireland; 2 Nimbus Centre, Munster Technological University, Bishopstown, Cork, T12 P928 Ireland; 3 School of Public Health, University College Cork, Cork, T12 K8AF Ireland

**Keywords:** Severe mental illness, Serious mental illness, Enduring mental illness, Public and patient involvement, Co-design, Co-creation, Digital physical activity intervention, Physical activity, Web-based application

## Abstract

**Background:**

Individuals with severe mental illness (SMI) engage in less physical activity (PA) and experience an earlier mortality rate than the general population. PA offers individuals with SMI multiple benefits, improving their physical and mental health. However, barriers are present that minimise the engagement of individuals with SMI in PA, including SMI symptoms, a lack of motivation, a lack of support and a lack of PA knowledge. Digital tools incorporated in PA interventions can assist in minimising these barriers and act as a long-term PA support for the SMI population. A gap remains in the literature on incorporating the voices of those with a lived experience of SMI in developing a long-term digital PA intervention. This project utilised a Public and Patient Involvement (PPI) approach in designing a digital web-based PA application for the SMI population in an Irish mental health residential setting, understanding SMI perspectives and influencing the design. The objective of this paper is to explore (i) the PPI process, and (ii) the impact of PPI.

**Main body:**

A local clinical psychologist was contacted to assist in identifying suitable contributors. Two individuals with a lived experience of a SMI living in an Irish mental health residential setting and two clinicians (i.e., stakeholder representation) working within these settings were identified. The individuals with a lived experience of a SMI and the clinicians were separated, and each group contributed to separate discussions. Each group participated in two discussions, sharing their ideas and views on the design of the web-based PA application. Each contributor provided insightful feedback on the design and development of the web-based PA application. Key highlights of the discussions involved the need for the web-based application to include more visuals in comparison to writing, suitable terminology, multiple PA options, multiple intensities, font type, layout design and the exclusion of a timer and a login feature.

**Conclusion:**

Based on the valuable feedback from the contributors, the web-based application was designed accordingly to address the needs and preferences of individuals with a lived experience of SMI and their clinicians working in the Irish mental health residential setting.

**Supplementary Information:**

The online version contains supplementary material available at 10.1186/s40900-025-00735-x.

## Abstract (plain language summary)

Individuals with severe mental illness often face barriers when trying to engage in physical activity. This can be due to symptoms of their mental illness, a lack of support or a lack of motivation. Adding a digital tool to a physical activity intervention for people with severe mental illness can help reduce these barriers and increase their physical activity. Using Public and Patient Involvement in research can improve the quality of the digital physical activity tool. By incorporating the voices of individuals with severe mental illness, this project can design a suitable digital physical activity tool that meets their needs, increasing their physical activity and long-term use. This project used Public and Patient Involvement to design a web-based physical activity application for people with a severe mental illness living in an Irish mental health residential setting. This paper will explore (i) the Public and Patient Involvement Process, and (ii) the Impact of Public and Patient Involvement.

A clinical psychologist helped find appropriate individuals willing to give their opinions and views on the design. These individuals included two people living in an Irish mental health residential setting with a lived experience of a SMI and two clinicians working within these settings. The individuals with SMI and the clinicians were separated into two groups. The groups participated in two discussions each. These individuals provided useful feedback on what elements to include and/or exclude in the design of the web-based PA application. Their valuable feedback helped shape the appropriate design of the web-based PA application and create this to suit the needs and preferences of the SMI population.

**Supplementary Information:**

The online version contains supplementary material available at 10.1186/s40900-025-00735-x.

## Background

A severe mental illness (SMI) is an umbrella term for a range of complex mental disorders, including schizophrenic, delusional, mood, neuroses, behavioural and personality disorders [1]. Individuals living with a SMI can experience delusions, depressive and/or manic moods, hallucinations and disorganised thinking [1, 2]. It is estimated that individuals with SMI have a shorter life expectancy, ranging from 15 to 20 years less than that of the general population [3, 4].

In addition to the mental health challenges faced by individuals with SMI, physical health is often compromised [4, 5]. Many individuals with SMI do not meet the World Health Organisation (WHO) guidelines for physical activity (PA), which recommend 150–300 min of moderate-intensity aerobic PA or 75–150 min of vigorous-intensity aerobic PA throughout the week [6]. Additionally, this population appears to be more sedentary than the general population, spending an average of 476 min per day in sedentary activities [6, 7]. In comparison, most adults in Ireland, without a SMI, are estimated to spend approximately 300 min per day in sedentary behaviour [8], highlighting a difference between individuals with and without SMI. Research indicates that addressing sedentary behaviour and increasing the PA among individuals with SMI can lead to multiple benefits, such as improved symptoms, higher quality of life and increased cardiorespiratory fitness [9, 10].

There are barriers to PA participation for those with a SMI, such as their mental health symptoms, lack of support, motivation, fear of injury and a lack of PA knowledge [11]. Digital PA interventions that increase PA have been shown to improve the mental and physical health outcomes of the SMI population [12, 13]. Digital tools can benefit the population, such as increasing their digital inclusion, self-management, motivation, empowerment and for therapeutic use (e.g. behavioural or psychotherapy) [14–16]. Digital solutions can assist in overcoming the PA barriers individuals with SMI face while offering a flexible and personalised approach to an intervention [17]. However, there is a gap in the research on the use of a personalised approach to long-term digital PA interventions for individuals with SMI, particularly in a mental health residential setting.

Personalisation is explained as tailoring the user experience of the product and/or service to meet the individual’s specific needs and preferences, with a mechanism of personalisation as offering the user a choice [17, 18]. Adapting a personalised approach can positively impact the individual’s satisfaction, accessibility, acceptability and uptake of the product/ service [19–23], therefore these benefits are often lost if personalisation is omitted. A previous study involving participants with haemodialysis observed declining attention and reduced motivation due to the lack of personalisation of a study component [24]. Moreover, concerning a long-term digital PA solution, the lack of a personalised approach in mental health residential settings presents further complexities. Challenges faced within these settings include time constraints of clinicians, staff shortages, complex mental health challenges, lack of service user engagement, limited access to facilities and resources and a ‘one size fits all’ approach [11, 25–27]. The staff shortages and time constraints may limit the clinician’s ability to personalise interventions to meet the service users’ specific needs [25, 28]. However, personalisation may be necessary to promote a healthy lifestyle and prevent chronic conditions [29]. Therefore, digital tools that incorporate a personalised approach can enhance their commitment, reliability and fulfilment, while also considering the barriers related to digital literacy [17]. Furthermore, the use of digital tools can reduce clinician’s workload [30] while acting as a long-term solution to increase PA for individuals with SMI.

Given the importance of personalisation, Public and Patient Involvement (PPI) emerges as a promising solution to ensure that the end user’s needs and preferences are met. PPI is defined as “research carried out ‘with’ or ‘by’ members of the public rather than ‘to’, ‘about’ or ‘for’ them’” [31] and has been recognised as a key element in creating successful digital tools for mental health populations [32]. By adopting a PPI approach to mental health research, it can progress in terms of how it is conducted, communicated and implemented, offering the SMI population a more personalised approach. Involving individuals with lived experience of SMI in planning and designing a digital PA application has the potential to increase its engagement and usability [31, 33].

While there have been positive developments in mental health research utilising PPI, particularly in co-designed PA interventions for individuals with SMI [34, 35], there remains a gap in the research regarding the use of PPI in developing a long-term digital PA intervention specifically for the SMI population in mental health residential settings. Recently, a ‘Digital for Care: A Digital Health Framework for Ireland 2024–2030’ was proposed, highlighting patient empowerment as the main influencer. There is optimism that providing individuals with enhanced access to digital services will yield significant positive impacts [36].

Given that PA levels among this cohort are low compared to the general population, there is a clear need for an effective solution to increase PA participation. Personalised approaches have been shown to be useful in addressing individual needs, while digital tools offer accessible and adaptable interventions. Therefore, this paper has two aims; to report on (i) the PPI process and (ii) the impact of PPI for developing a web-based PA application for individuals with SMI. Firstly, it describes the process of involving two individuals with SMI living in an Irish mental health residential setting, alongside two clinicians working in the same setting (as stakeholder representatives), in developing a web-based PA application for individuals with a SMI and their clinicians. Additionally, the second aim of this paper will include the impact of their involvement by presenting their perspectives on (i) PA barriers and facilitators, (ii) digital knowledge and (iii) digital web-based PA application feedback and how their perspectives shaped the web-based application that was developed for feasibility testing.

## The PPI process

This section outlines the procedure for using a PPI approach to design and develop the web-based application. It includes using the ‘Move More’ toolkit, how contributors were identified, and the content of the two discussions.

### The Move More Toolkit

This research, in collaboration with Loughborough University (United Kingdom (UK)), utilised the co-designed ‘Move More Toolkit’ [37]. The toolkit was co-designed by researchers at Loughborough University in collaboration with individuals with SMI and staff from an inpatient psychiatric ward [37]. This toolkit is an original, co-designed physical PA resource guided by the theoretical COM-B model, which addresses the themes of capability, opportunity and motivation [38]. The toolkit was developed specifically for individuals with SMI in a secure psychiatric unit in the UK and their clinicians to support the SMI individual in engaging in PA (supplementary materials 1, 2). The decision to digitalise the ‘Move More’ toolkit aimed to increase the cohort’s accessibility, offer choice, improve digital literacy, and enhance support and motivation while embracing innovation, engaging with the evolving future of a digital world and reducing clinician workload. A PPI approach involving individuals from this Irish setting was necessary to ensure the digital adaptation of the ‘Move More’ toolkit was appropriate for use in an Irish mental health residential setting. Through PPI discussions, this theoretical resource was developed into a digital web-based application aimed at individuals with SMI and their clinicians in an Irish mental health residential setting. The web-based PA application is designed to help individuals with SMI engage in PA, while also being used as a valuable resource for clinicians to support their service users in participating in such activities. A web-based application is an application program stored on a remote server and accessed through the internet (i.e. a web browser). This platform is free and accessible across multiple devices (e.g. desktop, laptop, smartphone) via a web browser, eliminating the need for separate application installation [39, 40].

### Identifying contributors

During the period February 2024 to April 2024, the primary researcher contacted a clinical psychologist who managed local mental health residential settings within one locality. The overall project’s purpose and the importance of utilising PPI within the project’s development were explained to the clinical psychologist. The clinical psychologist, who recognised the value of their role, agreed to identify contributors for the project. They then contacted clinicians and service users within one of their residential settings, stimulating the interest of two clinicians and two service users.

### Contributors

The contributors involved two clinicians working within a mental health residential setting (i.e., clinical nurse manager and mental health student nurse) and two service users with a lived experience of SMI, residing in the mental health residential setting. The service users were aged between 40 and 50 years old; one identified as a woman and the other as a man. The length of stay for the two service users differed; one had been residing in the setting for seven months, while the other had been there for three years. Both clinicians identified as women; one had been working in the setting for 16 weeks and the other for six years.

### Conducting the discussions

The clinicians and the service users were divided into two groups: group one consisted of the clinicians and group two included the service users. Each group participated in two separate PPI discussions, lasting 45 min to 1 h. Both discussions included a proposed agenda outlining the topics for discussion (supplementary materials 3, 4). The primary researcher acted as the facilitator. Although this was their first experience facilitating PPI discussions, they had completed online training (i.e., Digital Badge in Public and Patient Involvement in Research and PPI Shared Learning Groups) and engaged in ongoing discussions with PPI experts before independently leading the discussions. Additionally, the researcher had prior familiarity with mental health settings through visits and experience living and working with individuals with additional needs. A key aim of these discussions was to encourage informal conversations and for ideas to flow freely among the contributors. The PPI process involved separate discussions with service users and clinicians. Although feedback from both groups was carefully considered by the researcher, a formal consensus process was not undertaken. In cases where perspectives differed, priority was given to service user input to ensure that the final features were aligned with their needs and authentically represented the lived experiences of individuals with SMI.

### Discussion one: introductions, what is PPI? And initial perspectives on barriers to PA in residential settings

The first discussion was a crucial step in the project’s development. It involved an introductory session, explaining the research focus, and outlining the importance of PPI to all contributors. This introduction helped the contributors feel at ease with the researcher and establish a rapport. Furthermore, the service user’s PA experiences and the barriers and facilitators they face within their residential setting were delved into through discussion using questions and word prompts. This aim was to understand these barriers and facilitators from their own perspectives, which may be similar to other individuals with SMI living in a mental health residential setting. We also explored the service users’ digital knowledge, familiarity with digital tools and what they find enjoyable about them. Additionally, conversations during the first discussion with clinicians focused on identifying what would encourage them to motivate service users to participate in PA.

The primary researcher utilised printed words and visuals to act as prompts throughout these discussions (supplementary material 5). This creative method in PPI has been shown to be successful in assisting contributors to get more involved, create a safe, open space to share and feel equal amongst the researcher [41, 42]. Lastly, the overall study methodology was shared to gather feedback on different aspects, including the intervention timeframe and data collection involving individuals with SMI, such as the use of accelerometers and interviews.

### Discussion two: the move more toolkit development into a web-based application

The second discussion occurred two weeks later and involved a more practical approach. Each contributor was presented with a physical copy of the ‘Move More’ toolkit. All contributors reviewed each page of the toolkit and engaged in discussions around the adaptation of the physical copy to a digital web-based application. These conversations included the use of appropriate terminology for an Irish mental health residential setting, colour scheme, layout, font type, font size, instructional videos and visuals. The contributors identified additional physical activities to add to the web-based application, including trialling out a game to determine whether it is suitable for the SMI population. The contributors were shown visual templates of different web-based application layouts to assess whether they would prefer a similar design on the web-based application (supplementary materials 6, 7, 8, 9).

### Recordings and note-taking

While ethical approval for PPI is not required [31] and audio recording is uncommon for PPI, ethical approval was sought and granted to accommodate this (Clinical Research Ethics Committee approval number: ECM 4 (w) 05/12/2023 & 5 [6] 05/12/2023 & ECM 3 (sss) 06/02/2024 and Munster Technological University ethical number: MTU-HREC-FER-23-039-A). The reason for the audio recordings was to allow the researcher to be fully present in the conversation. Additionally, the researcher wrote detailed notes and transcribed the discussion to ensure that all participant’s contributions were captured. All the contributors agreed to these recordings before the beginning of each discussion.

### Reimbursement for contributors

After discussion two was completed, each contributor was gifted a One-For-All shopping voucher as a token of gratitude for dedicating their time to feedback, giving their opinions, and sharing their perspectives on the different areas.

### GRIPP2-SF

To record the involvement of PPI in this work, the GRIPP2-SF (Guidance for Reporting Involvement of Patients and the Public- Short Format) was adhered to (supplementary material 10) [43].

## PPI impact

This section outlines the impact of PPI, which influenced the design and development of the web-based PA application. This is divided into three main themes identified from the discussions: (i) PA barriers and facilitators, (ii) digital knowledge, and (iii) the key feedback developed for the digital web-based application and how this was incorporated into the digital tool.

### PA barriers and facilitators

We sought general feedback on the barriers that individuals with SMI perceive when participating in PA. Service users did not mention any barriers to engaging in PA. According to clinicians, the most evident barrier to PA participation for service users is the risk assessment conducted by clinicians, which reduces the service users’ independence. Additionally, clinicians recognised the service user’s lack of motivation as a barrier to PA participation. Clinicians mentioned that losing their occupational therapist has resulted in less PA engagement. The clinicians stated that *“it can be difficult” to* encourage service users to partake in PA, and *“it can fall to the back of your mind…when you’re doing your own jobs”*.

Discussions around the space within the area outlined that the clinicians felt they *“could do with more”.* The clinician recalled an exercise class where she had to facilitate it in the sitting room, where only three people could join, and the area was not large enough for more to participate. On the contrary, the service users felt they had enough space to participate in PA around the residential setting.

The clinicians mentioned that SMI side effects can act as a barrier to PA for this cohort. Issues related to medications, weight gain and appetite can negatively impact the desire to participate in PA. Additionally, clinicians spoke about individual capabilities for SMI PA participation, stating that some individuals with SMI might independently participate in PA, whereas others would need more prompting and motivation.

Questions were asked to all contributors about what they felt helped the service users to participate in PA. The service users regarded clinician support as a key facilitator of their PA engagement, as the clinicians ‘*would take us out*,* and we’d go for a short walk’.* The service users emphasised that these walks may be in a group setting and a new location every time. These walks are seen as a social outing to meet other service users and to enhance social skills. Additionally, it was mentioned that individuals are not forced to partake in group walks if they wish to walk individually. Service users highlighted the motivational element of participating in these walks, as they could go for a coffee afterwards. This highlights the social and psychological value of PA. However, the clinicians expressed concern that the food element, acting as a reward for service users, might undermine the health benefits of the PA, particularly given the high risk of weight gain and sedentary behaviour in this population.

The setting facilities, both on-site and off-site, were seen as facilitators of PA participation. The service users spoke about the town being within walking distance from the setting, as well as a walking track outside and weekly pitch-and-putt, which they attend regularly. The walking track was highlighted throughout the discussions and seems popular among service users. All contributors highlighted the gym equipment that they had within their setting, such as bikes and rowing machines. However, a clinician stated that due to the facilities and available space within the setting, they could only facilitate certain activities to a limited extent. A PA routine was also seen as a big facilitator for service user PA participation. The clinicians emphasised that once a service user does PA routinely, they tend to pick it up and build it into their daily routine.

### Digital knowledge

It was noted that digital knowledge depends on the service user and their capabilities to use digital tools. The setting does provide tablets to service users, with service users stating that they use them sometimes. Clinicians mentioned that some service users might need little support to help them use the tablet. However, most service users would have the capability to use the digital tools themselves. When asked further about the service user’s digital knowledge, the clinician clarified, *“It would be*,* they’d have digital health knowledge*,* but it’d be limited…they would need to be shown how to use the app. But…once they were shown*,* they will be able for it”.* This clarification reassured the researcher by showing the potential for a web-based application in a residential setting.

### Digital PA web-based application feedback

#### Multiple PA options and intensities

The contributors expressed appreciation for all of the physical activities included in the original physical copy of the ‘Move More’ toolkit. All these physical activities were retained for the web-based application. However, the contributors reviewed and considered the inclusion of different physical activities. All the contributors suggested retaining the gym workouts in the application, as they provide educational value to SMI participants, even if the setting does not have a gym, *“I’d rather have it in*,* rather than not have it in… It’s a bit of education…”.* There were also recommendations to ensure that some of the existing physical activities would be more descriptive than what was originally presented (e.g. shadow tag). Additionally, suggestions were made to incorporate more diverse physical activities, such as dancing, group games, alternative walking and additional physical activities from different resources (supplementary material 11), all of which were selected for inclusion in the web-based application.

The discussion concerning the motivation of the PA web-based application was a key component of the intervention. The clinicians favoured the inclusion of multiple physical activities in the web-based application as it would provide choice for the service users, making them more willing to do some PA. Furthermore, clinicians noted their role in encouraging service users to utilise the PA web-based application. This encouragement would assist service users in eventually building it into their daily routine and, thereafter, using it independently.

In relation to PA intensities, all contributors agreed that the web-based application should include multiple PA intensities. This was argued to accommodate SMI participants who might need to begin with a lighter intensity and build up to a moderate intensity. Additionally, the suggestion was to allow all SMI participants to benefit from the addition of multiple intensities and to provide them with a choice, which may increase their motivation to participate in PA. This feedback was incorporated into the web-based PA application. The majority of physical activities included are light PA intensity (e.g., a chair-based workout). However, individuals with SMI can choose a moderate PA intensity (e.g., a 5-minute circuit). The addition of more physical activities, including those with greater intensity, will be reassessed after the initial phase. Additionally, the web-based application will include further education resources for SMI participants, such as (i) the benefits of ‘moving more’ (ii) barriers to ‘moving more’ (iii) myths and (iv) resources on how to be active. These resources have been adapted from the original ‘Move More’ toolkit. All contributors agreed to include these elements on the web-based PA application.

#### Terminology and visuals

The simplicity and ease of use of the web-based application were emphasised as key components for its success. Clinicians stated that certain service users might struggle with literacy; therefore, simple language, free from sensitive terminology, was deemed essential to meet these barriers. Similarly, service users favoured a design with more visuals than text, as the visuals were considered more suitable for helping them associate and understand activities. Additionally, it was suggested that videos would be easily explained. However, videos were deemed unnecessary for all physical activities and should only be included for more challenging physical activities (e.g. burpees, squats, etc.) to aid understanding. By adopting this visual-centred, straightforward approach, the web-based application can reduce complexity. This, in turn, assists clinicians in encouraging service users and helping them to become more independent when navigating the web-based application. Based on this feedback, this resulted in incorporating simple language and visuals throughout the web-based application. The web-based application has inserted more visuals than text. As per the feedback, the videos included in the web-based application were for the more complicated ones. However, more videos were included for the physical activities that may not be clear to service user from a picture.

*“If they do have difficulty with reading*,* writing*,* if there’s a picture there that they can associate*,* might make it a bit easier” (Clinician).*

The discussions around the PA instructions within the web-based application led to the decision that a guided approach (i.e. “*you see someone raise your hand*,* and then they raise their hand kind of a way”*) would be more beneficial for the cohort than a numbered, step-by-step approach (i.e. step 1, step 2). The guided approach was seen as less demanding and would likely put the participant at greater ease when engaging in PA, making the overall experience comfortable and encouraging. The researcher aimed to ensure that all physical activities on the web-based application followed this approach.

The terminology from the original physical copy of the ‘Move More’ toolkit was explored among clinicians and service users. These terminology discussions helped shape the language for the digital version aimed at an Irish mental health residential setting. Service users expressed a preference to be referred to as ‘service users’ instead of ‘patients’. Moreover, clinicians preferred to be referred to as ‘clinicians’ rather than ‘staff’. The contributors agreed to replace the term ‘ward’ with ‘unit’. This terminology discussion was required to reach individuals with SMI from diverse backgrounds and mental health histories, allowing for a more respectful and inclusive approach. The careful selection of suitable terminology reduces the likelihood of offending individuals with SMI and/or clinicians. The terminology was a vital element of the web-based application and was essential to be discussed with the contributors. Therefore, using the chosen terms was incorporated into the web-based application.

Additionally, the headings from the original ‘Move More’ toolkit were reviewed, leading to a decision to merge headings such as ‘outdoor activities’ and introduce new headings such as ‘group games’ to better reflect the expanded scope of physical activities. These headings were included on the web-based application alongside other headings to add such as, light intensity PA and moderate intensity PA.

#### Resources and a login feature

Downloadable PDF resources were deemed the most suitable for service users and were integrated into the web-based application. *“I could see them doing pen and paper. But I can’t see them logging online… If it was maybe a printable… could see them using the app and writing down what they did”.*

The implementation of a login feature was identified as a potential barrier to the web-based application. This concern arose because the majority of service users do not have an email address, making the login process a possible obstacle for them. Additionally, the possibility of losing the physical activities sword was also deemed a potential barrier. As a result, it was suggested that the web-based application exclude a login feature. This approach would eliminate potential barriers, making the web-based application more accessible for service users. The login feature was not included in the web-based application.

#### Web-based application layout

All participants were satisfied with retaining the original colours and font style in the web-based application, maintaining consistency with the physical version. The original colours were adapted into the digital version, and the researcher aimed to keep a similar text style. In addition, discussions were held around incorporating motivational quotes into the web-based application. The SMI contributors agreed that such quotes could *“spur a person on”* and enhance user engagement. Due to this feedback, a motivational quote was added to the home page of the web-based application.

Opinions were divided when discussions were held about adding a timer to the physical activities. Clinicians supported the inclusion of a timer, seeing no harm and believing it could encourage service users. However, service users disagreed, expressing concerns that people complete different physical activities at different paces, making it difficult to gauge a universal timer that suits all service users. As a result of that feedback, the decision was made to exclude the timer. However, the inclusion of a timer will be revisited and reassessed during the next round of PPI feedback.

Table 1, below, is a visual representation of the topic discussed, what was implemented within the web-based application, and how this was decided.

**Table 1:** Discussion topics & application of feedback

PPI Discussion Topic	What Was Implemented within the Web-Based Application?	How This Was Decided
Motivational quotes	Addition of motivational quotes.	The facilitator questioned the contributors whether motivational quotes would be useful within the application. All contributors agreed that this can enhance user engagement and wished for the web-based application to include them. The service users appeared to enjoy the idea of motivational quotes in the web-based application.
Physical activity options	All the physical activities in the ‘Move More’ toolkit, including the gym workouts, were retained. Different physical activities were added, including dancing, group games, alternative walking, etc.	Contributors reviewed each activity from the toolkit and agreed to keep them all. They then brainstormed additional physical activities that were commonly enjoyed in their setting and that may be useful to add. The facilitator also presented a PA resource with various physical activities. The contributors trialled and selected suitable physical activities for inclusion in the web-based application.
Physical activity intensity	Light PA intensity and moderate PA intensity workouts were included.	Contributors agreed to include light PA intensity options on the web-based application, but also include moderate PA intensity to be able to reach a wider range of individuals with SMI who may wish to do a more intense workout.
Educational resources	The inclusion of (i) the benefits of ‘moving more’, (ii) barriers to ‘moving more’, (iii) myths, and (iv) resources on how to be active. All from the original ‘Move More’ toolkit.	The contributors reviewed the original education elements in the ‘Move More’ toolkit and wished to retain them in digital form.
Terminology	The terminology decided to include (i) unit, (ii) service user, and iii) clinician. The terminology was kept simple and clear.	Both groups discussed the terminology. Service users and clinicians shared preferences on how they wished to be referred to in the application. The contributors discussed the need to avoid stigmatising and confusing terminology, opting for simple language on the web-based application.
Visuals (i.e., pictures and videos)	More visuals were inserted into the web-based application than text. The visuals included pictures guiding the PA. Videos were included for the more complex physical activities and physical activities that may not be easily understood from a picture.	All contributors were in mutual agreement that having more visuals on the web-based application would be more accessible and easier to follow compared to text. A service user specifically recommended videos for activities that are difficult to understand through text or pictures alone.
Downloadable PDFs	Downloadable PDFs were added to the web-based application.	Contributors appreciated the resources included in the ‘Move More’ toolkit and suggested downloadable versions to print for service users.
Login Feature	The login feature was excluded.	Contributors felt the login feature would be a barrier, as most individuals with SMI do not own a digital tool or an email address. To maximise accessibility, an agreement was reached to exclude this feature.
Original colours and font style	These were maintained from the physical ‘Move More’ toolkit and included in the digital version to the best of our ability.	The contributors enjoyed the ‘Move More’ toolkit’s colours and font, and were happy that the web-based application looked similar.
Timer	Excluded from the web-based application.	The facilitator questioned the use of a timer for the web-based application. The clinicians agreed that a timer should be included; however, the service users disagreed, feeling it could add pressure. To respect service user input, this has been excluded, with the option to revisit it during later stages of retesting.

## Reflections on the process and the impact of PPI

This paper aimed to report on the PPI process in this project and describe its impact on developing a web-based PA application for individuals with SMI. Through the involvement of individuals with SMI and clinicians working in an Irish mental health residential setting, the project incorporated perspectives that informed the design and development of the web-based application, helping meet the needs of individuals with SMI.

### Involving individuals with SMI and their clinicians in shaping the web-based PA application

The involvement of the demographic profiles of the contributors impacted the outcomes of the web-based application. The contributors were specifically individuals residing in a mental health residential setting living with SMI. Additionally, clinicians working with these populations were also targeted. This was due to the web-based applications being tailored for individuals with SMI and clinicians working in a similar setting. Their perspectives from an analogous setting assisted in defining key elements of this web-based application. This ensured that the design was focused and met the needs and preferences of the individuals. A negative stigma attached to this cohort is apparent [44]; therefore, focusing on individuals from SMI backgrounds is essential to minimise this situation and involve an inclusive approach. The contributors were encouraged to openly share their views on different aspects of the research design. For instance, discussing the terminology used in the web-based application is crucial because choosing terms appropriate for the intended audience can help reduce stigma and promote empowerment [45, 46]. This PPI process enabled the participants to feel empowered, which was fostered within the project [45, 46]. It has been mentioned that there has been a considerable lack of PPI reporting [47]. Therefore, this paper addresses this gap and mentions specifically how the contributors were involved and the results of their involvement [43], adding to current research in the field.

### Impact of PPI on the intervention

Based on the feedback provided in discussion two, the web-based application was designed to reflect these. The researcher prioritised closely adhering to contributors’ feedback, ensuring that the design remained aligned with their discussions. This feedback was instrumental in shaping the web-based application to meet the needs of individuals with SMI residing in a mental health residential setting in Ireland. The initial objective of PPI was to create a suitable digital tool for the cohort, and the insights gathered, greatly influenced its design. Notably, the contributors wished for the digital version of the toolkit to be accessible and engaging for individuals with SMI. They offered feedback on different aspects, such as the inclusion of additional physical activities, PA intensities, visuals, suitable terminology, printable PDFs and the exclusion of a login feature.

Visuals (i.e., pictures and videos) emerged as a key inclusion in the design of the web-based application as this would improve engagement and usability. The contributors expressed a clear preference for the inclusion of visuals in comparison to ‘heavy’ text instructions. Literature has found that providing instructions on how to perform PA and access to online resources for additional physical activities are effective and engaging elements in digital technology for individuals with chronic conditions [48]. These features align with behaviour change techniques [49] and support the delivery of self-guided interventions [48]. By incorporating visuals into the web-based application, this enhances accessibility and empowers the service user to engage with the web-based application independently.

The contributors explained that the inclusion of multiple physical activities and PA intensities within the web-based application would be helpful as it can accommodate a variety of individuals with SMI, regardless of their PA abilities. Without PPI feedback, the researcher may have alluded to certain physical activities and one intensity, limiting the choice for all SMI participants. Moreover, contributors felt the educational elements from the ‘Move More’ toolkit should be incorporated into the web-based application. By providing the education elements within the web-based application, the participants not only enhance their understanding of the importance of being physically active [37] but are also provided strategies to overcome these obstacles. This comprehensive approach can empower SMI participants, showing multiple ways to be physically active. The educational element provides a supportive resource that benefits both service users and their clinicians.

Choosing suitable terminology was an essential focus of the PPI discussions, as stigmatising and negative terminology can harmfully affect an individual with SMI’s self-esteem and personal goals [50]. The researcher approached this discussion cautiously, as they did not want to upset anyone. Through reflection and open dialogue, contributors highlighted the importance of using familiar, respectful, and appropriate terminology within their setting. Their insights informed the language used within the web-based application. By selecting suitable and familiar terminology, this ensured that the web-based application would be user-friendly, which may help mitigate barriers and increase their engagement with the PA web-based application.

The contributors provided useful advice regarding the inclusion of a login feature. Given that this population experiences digital exclusion [51], it was agreed that the removal of a login feature was deemed applicable, making the web-based application more accessible to reach the wider SMI population and not exclude anybody. Furthermore, the resources from the ‘Move More’ toolkits were advised to be available in printable PDF format. The PDFs will allow users to download, print, and engage with the materials as they would not be able to write directly on them on the web-based application. The decision to remove a login and offer downloadable PDFs reflects the contributor’s feedback and emphasis on flexibility and accessibility for individuals with SMI.

The meaningful involvement of individuals with SMI and clinicians helped ensure that a suitable and relevant web-based PA application was developed for the cohort. By listening and incorporating their needs and preferences, the web-based application has greater potential to encourage and support PA engagement and help maximise the additional health benefits PA can offer this cohort.

Overall, while the design of the ‘Move More’ toolkit maintained its core elements (e.g., layout, colour, font, physical activities, resources), the PPI discussions introduced meaningful adaptations to improve it into a web-based application. Feedback from contributors was vital to the design and further development of the web-based application. This reflected the perspectives of individuals living with a SMI and included representation from the clinician stakeholders involved in their care. Without this influential feedback, the researcher may have limited the web-based application and not met the cohort’s needs. This demonstrates the value and impact of PPI in the design and development of a web-based application aimed at the SMI cohort.

Future directions of this project involve feasibility testing of the web-based PA application to evaluate its effectiveness, acceptability and benefits for individuals with SMI. Insights from this evaluation will inform further improvements to the web-based application to enhance its relevance for the cohort.

### PPI process

Identifying contributors was a time-consuming aspect of the process, as similarly noted in a previous study [52], particularly when it comes to identifying participants for mental health research [52, 53]. Researchers should allow sufficient time to identify contributors, as reaching this population can be challenging. This can be due to ethical issues, busy working conditions, privacy concerns, mental health symptoms and a lack of awareness of the importance of PPI in research. Therefore, the researcher ensured sufficient time was allocated to identifying SMI contributors and their clinicians, conclusively helping the researcher to save time and maintain progress with the research schedule.

Furthermore, while our engagement within the PPI discussions was fruitful, it is important to note that the researcher approached contributors with a previously designed toolkit (i.e. the ‘Move More’ toolkit). Although the toolkit was a co-designed physical copy, this may have restricted the opportunity for contributors to contribute their ideas and suggestions from the beginning. The PPI process may have been more impactful if it had allowed the contributors to create the web-based application from the foundation rather than having a perceived idea. However, having the physical copy of the ‘Move More’ toolkit and web-based application layout ideas, enabled contributors to better visualise how the web-based application might appear. This aimed to empower contributors to contribute more effectively. Additionally, although the combination of group discussions, word prompts, layout ideas, and the physical copy of the ‘Move More’ toolkit was time-consuming, this allowed for more in-depth valuable feedback, which was critical for the success of the PPI discussions. Future researchers should consider providing visuals to maximise the effectiveness of the discussions, as this can help convey ideas more clearly and encourage greater engagement [41, 42].

The facilitation of how the PPI discussions were conducted was a strength of this process, as it ensured the contributors were engaged in an inclusive way, influencing the development of the web-based application. The location where the clinician worked and the individuals with SMI resided were suitable for the discussions. This facilitation allowed the individuals with SMI and clinicians to participate in the PPI discussions with flexibility and ease, without the necessity of arranging transportation or consuming additional time. These considerations should be established prior to any PPI facilitation, allowing the contributors to share their recommendations on where best to facilitate these discussions.

It was decided to split the clinicians and service users into separate PPI discussion groups. This ensured each contributor was comfortable openly sharing their honest perspectives without conflicting views. Service users may be more comfortable sharing their ideas without a clinician present in the room. A prime example of this conflict was when the timer discussion arose in the PPI groups. The service users could openly voice their opinions based on their lived experiences and what they felt worked best for them, which was the exclusion of the timer. No contributor commented on the separation of PPI groups. Additionally, this separation allowed a more tailored focus discussion with each contributor. The discussions with service users were focused on their lived experience perspectives and what they would enjoy in the web-based PA application. Meanwhile, the clinician discussions focused on how they could use the web-based PA application to support service user participation in PA. While the discussions were similar, each was slightly tailored to address the unique perspectives of each group. However, this separation means collaboration between clinicians and service users did not occur. As a result, there were limited opportunities for sharing ideas and finding a mutually beneficial outcome (e.g., the timer discussion). While this approach supported open dialogue, it highlighted the limitations of conducting separate group discussions.

It is considered essential to acknowledge the contributors for their value, time, and perspectives in the project; therefore, it is good practice to reward/ reimburse them [31]. The contributors were rewarded with a €100 One For All shopping voucher as an appreciation for their effort in the discussions. The research team thanked all contributors for sharing their valuable ideas and perspectives. The researcher hopes to revisit the setting with the finished product. Studies should consider allocating funds for rewards/ reimbursement of certain costs to help ensure that engaging in PPI does not have any negative implications for the contributor and does not act as a barrier to attendance [54]. Future studies should recognise their contributors in some form of compensation, such as funding travel, childcare or vouchers.

Involvement in research can be challenging [55–57]. This PPI process is limited by the relatively small group of contributors, with two individuals with SMI and two clinicians partaking in separate groups. We aimed to engage as many individuals with SMI lived experience as contributors and their clinicians as possible. The small group limits the recognition of diverse populations with multiple perspectives. Furthermore, there was a reliance on the four contributors to provide feedback on the web-based application that would represent additional individuals with SMI and clinicians. The PPI could have benefited from including additional contributors to represent a broader range of perspectives and knowledge, potentially enhancing the diversity of insights. However, recruitment challenges arose due to the vulnerable nature of the population and difficulties in accessing them. Additionally, clinicians’ perspectives were relied upon when service users were unable to engage with or respond to certain questions due to the nature of their SMI. Therefore, clinicians provided input based on their knowledge and experience caring for individuals with SMI. In future studies, researchers should aim to recruit contributors in collaboration with clinicians and associated services to help identify potential SMI PPI contributors. In spite of this limitation, the four contributors provided extremely valuable feedback and may have felt more comfortable engaging due to the smaller group sizes.

Moreover, while we gathered valuable insights from our PPI groups during their two discussions, an area for improvement is facilitating more sessions. This limited number of discussions may have resulted in missing additional opinions, ideas, and perspectives from the contributors, which could have enriched the development of the web-based application. On reflection, additional discussions may have allowed for building a more meaningful rapport and for the contributors to speak more openly and freely [58]. However, a limited amount of PPI may be sufficient and provide a greater impact than a study that omits to include it, therefore enhancing the relevance and quality of the study, enriching the overall research [31, 59]. In future studies, researchers should allocate as much time as possible for effective PPI feedback, ensuring a friendly rapport is established. However, this project contributes to the ever-growing field of digital health for the SMI population and to the growing body of research on PPI studies by highlighting the importance and value this approach can offer.

## Conclusion

This paper aimed to show how PPI was effectively used in the design of a digital PA web-based application that would meet the needs and preferences of individuals with SMI residing within an Irish mental health residential setting. The collaboration with individuals with SMI and their clinicians greatly influenced the design appropriately by addressing elements such as (i) multiple PA options and intensities, (ii) terminology and visuals, (iii) resources and a login feature and (iv) the web-based application layout, ensuring an applicable and effective outcome.

While feasibility testing is currently ongoing, the findings from the PPI process demonstrate that involving those with lived experience of SMI can enhance empowerment, accessibility, usability and provide them with a meaningful voice in the process. This process will be particularly beneficial for those seeking guidance on initiating future PPI processes in their own research. Given the lower levels of PA and the increased risk of poor physical health among individuals with SMI, there is a need to develop a relevant and engaging PA intervention that meets the needs of this population. Including a PPI approach in the design of an intervention can ensure that they are meaningful and appropriate to the needs of the intended cohort and ensure better engagement. Future studies, particularly SMI research, should involve those with lived experience of SMI, to ensure the development of interventions are practical and effective for the cohort.

## Supplementary Information

Below is the link to the electronic supplementary material.

Supplementary Material 1

Supplementary Material 2

Supplementary Material 3

Supplementary Material 4

Supplementary Material 5

Supplementary Material 6

Supplementary Material 7

Supplementary Material 8

Supplementary Material 9

Supplementary Material 10

Supplementary Material 11

## Data Availability

No datasets were generated or analysed during the current study.

## Abbreviations

SMI: Severe mental illness
PA: Physical activity
PPI: Public and patient involvement
