# Supplementary material for: The role of public and patient involvement in designing a web-based, physical activity application for individuals with severe mental illness
Source: Res Involv Engagem. 2025 Jul 21;11:86. doi: 10.1186/s40900-025-00735-x (PMC12278672; doi:10.1186/s40900-025-00735-x)
Supplement: Supplementary file 2 — Supplementary Material 2 [file 40900_2025_735_MOESM2_ESM.pdf]

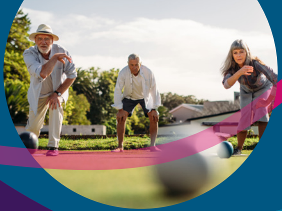

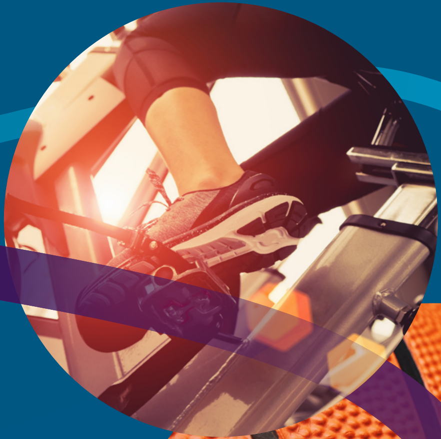

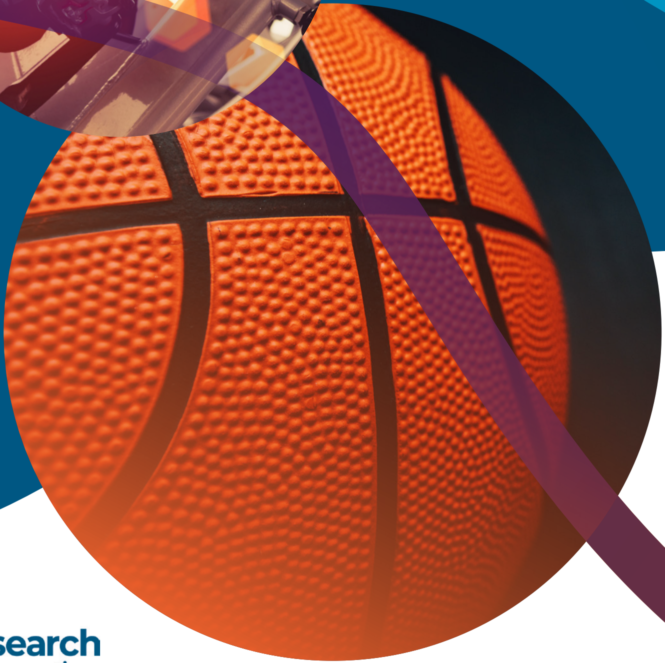

THE ‘MOVE MORE’ TOOLKIT

A STAFF GUIDE

A resource providing education, support and guidance for how you can help patients move more in secure care

Loughborough University

National Centre for Sport & Exercise Medicine
Working for Health & Wellbeing

Research & Innovation
Part of St Andrew's Healthcare

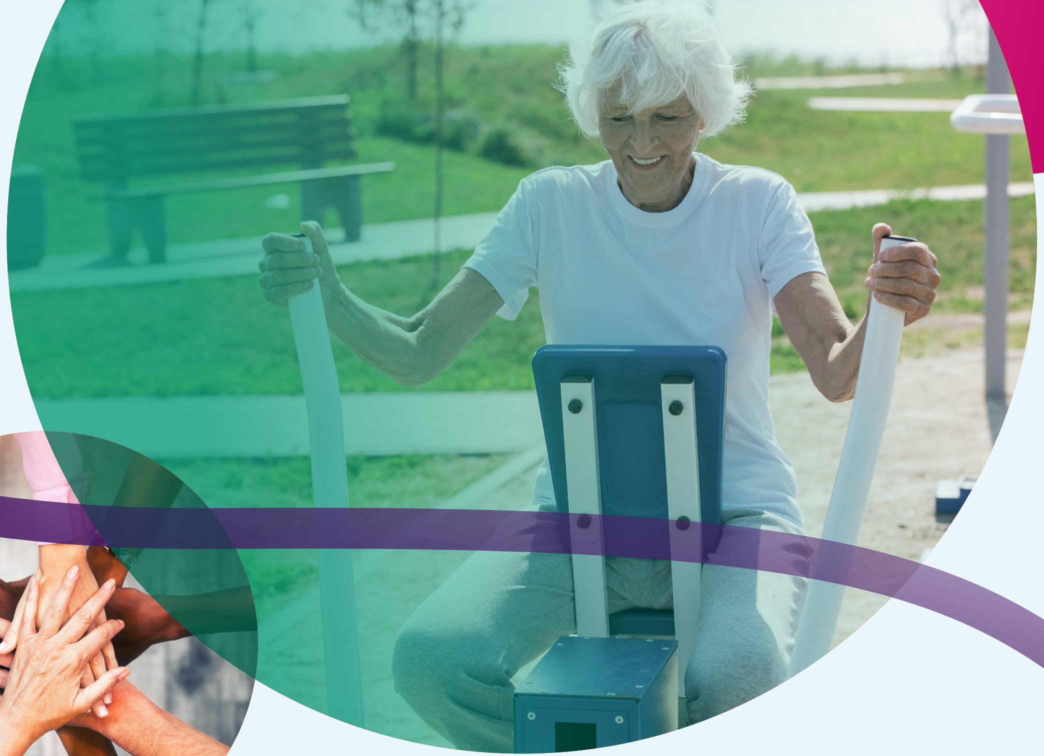

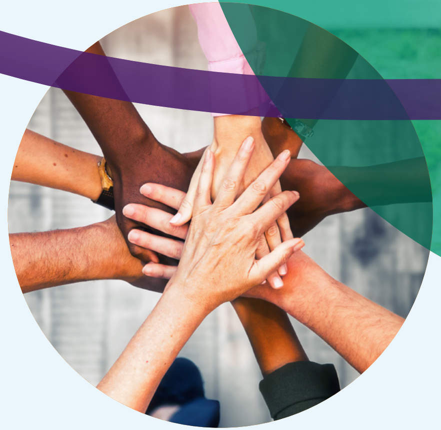

CONTENTS

1: OVERVIEW

2: MOVEMENT AND MENTAL HEALTH

3: HOW TO SUPPORT ACTIVITIES

4: ACTIVITIES

5: RESOURCES

THERE ARE 4 MAIN SECTIONS IN THE TOOLKIT

1. EDUCATION ABOUT THE IMPORTANCE OF MOVING MORE
2. PRACTICAL ADVICE FOR HOW YOU CAN SUPPORT PATIENTS TO MOVE MORE
3. IDEAS OF ACTIVITIES THAT CAN BE DONE WITH PATIENTS TO INCREASE MOVEMENT
4. RESOURCES

Aims of the toolkit

With this toolkit we aim to...

Increase **CAPABILITY:** ensuring patients have the appropriate physical skills, alongside the appropriate knowledge to be active.

Increase **MOTIVATION:** this relates to the decision making process involved in whether to choose to engage in a particular behaviour.

Increase **OPPORTUNITY:** this means looking at factors in the external environment such as time, location and resources which impact opportunities to be active.

It has been suggested that to perform a particular behaviour, patients must have the psychological and physical capability to do so. They must have enough opportunities to do the behaviour and they must be motivated to that behaviour

SECTION 2:

MOVEMENT AND MENTAL HEALTH

This section covers some basic information about the benefits of 'moving more'.

This section also gives guidance and tips for how you can talk about, and support patients to 'move more'.

What does being active mean?

Being active doesn't always mean doing structured sport and physical activity. There are many ways that patients can be more active.

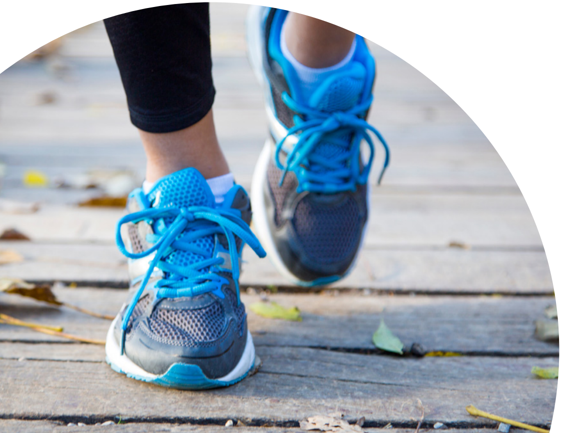

- A 5-minute circuit on the ward
- Breaking up times of sitting with standing
- Going to the gym, pool or sportshall
- Playing games on the ward
- Having a walk in the grounds
- Walking around the courtyard

Benefits of moving more for patients

- Improved Energy Levels
- Reduced Stress
- Better management of mental health symptoms
- Improved Cognition
- Management of physical health conditions
- Better Sleep

Physical health benefits of moving more

- Better management of the symptoms of diabetes by helping to control blood sugar levels
- Better management of asthma by increasing lung capacity and health
- Helps maintain a healthy weight, if needed
- Improved muscle strength and flexibility

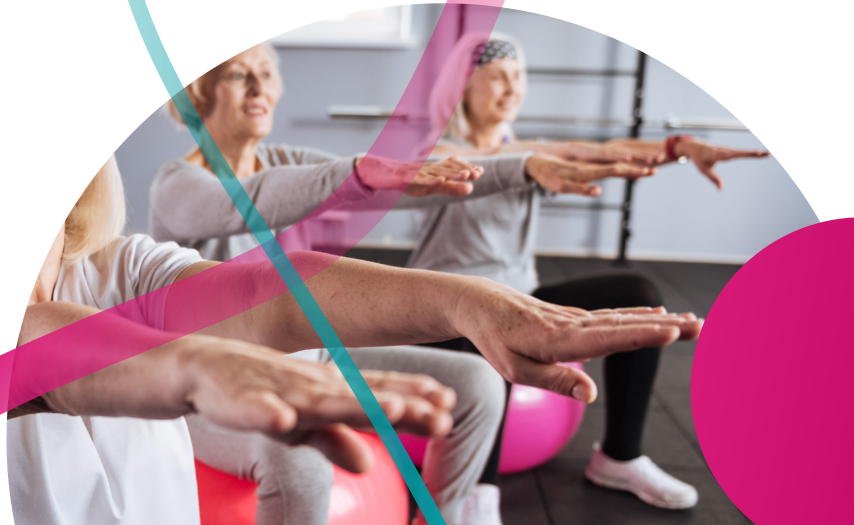

Despite the benefits of physical activity, some patients can find it challenging to be active. Here, we detail some of the common barriers that patients face to physical activity participation and gives some tips and guidance about how you can address these.

Common barriers	How you can help address these.
Motivation	Motivation levels for activities will fluctuate alongside fluctuations in mental health. If a planned activity is refused you could suggest a yoga sequence (page32) /5 minute circuit (page34) or maybe just a lap of a courtyard
Fatigue and tiredness	Fatigue and tiredness are common side effects of many medications. Ask patients when they feel most tired/alert and what time of day is best for them. This may differ depending on when patients take medication.
Poor physical health	Some patients will have physical health conditions that can make some physical activity difficult. It is important that you talk with the patient to work out the challenges they face and what types of activities might be beneficial.

Mythbusters!

This section lists some of the common myths about physical activity for patients in secure care

Myth	Fact
✗ *I need to be qualified to help patients be active*	✓ *While some activities will require the support of an exercise professional, basic activities can be supported by any member of staff.*
✗ *Physical activity has to be high intensity to be beneficial*	✓ *The most beneficial type of physical activity is one that is enjoyed by the patient. Many of the benefits of increasing movement occur regardless of intensity.*
✗ *Patients may be too unwell to be active*	✓ *Whilst poor mental and physical health can make it difficult to take part in some activities, some patients may benefit from doing more activities.*
✗ *Patients need to have leave to be active*	✓ *Whilst some physical activity can be done off the ward, there are many activities that you can do on the ward or in the courtyard, or even from a chair!*

SECTION 3:

HOW TO SUPPORT ACTIVITIES

This section gives practical tips and advice for how you can motivate and support patients to 'move more'

Before an activity: How to talk about moving more

- Ask what the patients would like to do more of – this will vary for every person. Consider the individual and listen to what they have to say.
- Ask what time of day they like to do activities. Some patients may take medication that may make them feel tired at certain times of day.
- Be aware that motivation and mental health can fluctuate which may impact capacity to engage in activities. If a patient refuses, always try offer activity again and don't give up.

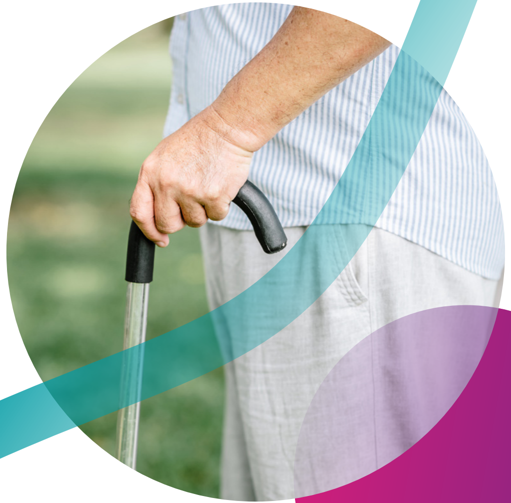

What to wear?

- Whilst different activities require different outfits, this provides a quick guide of the type of clothing that might be best to do physical activity in. The most important thing is that you are comfortable and feel you are able to move easily in the clothing that you are wearing.
- **Top:** A lightweight t-shirt or sweatshirt. If you go outside, consider wearing layers that you can take off if you get warm.
- **Bottom:** wear something flexible with an elastic waistband. This could be jogging bottoms, leggings or shorts.

Footwear: a pair of trainers that are comfortable and supportive.

During an activity:
How to support activities

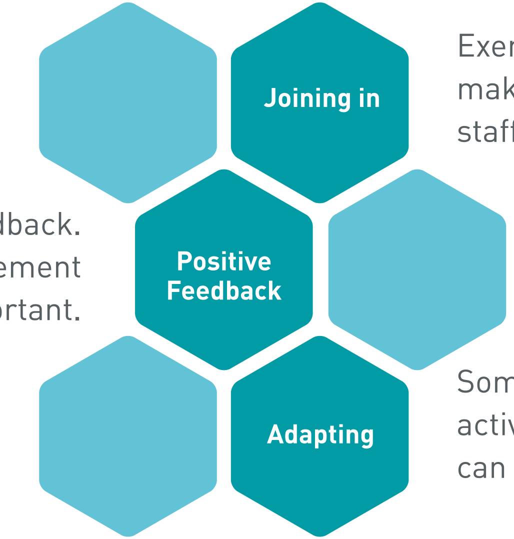

Exercising together boosts motivation, making the session more enjoyable for staff and patient!

Giving positive feedback. Support and encouragement is really important.

Sometimes you can't always do the activity you planned. Consider how you can adapt the time, place, duration.

After an activity

Praise the patient and say well done. Choose something specific to focus on with this praise.

Ask the patient if they enjoyed the activity and if they have any feedback. How could it made more enjoyable in the future?

See if the patient would like to record their activity anywhere (check the resources section for ideas).

Consider a certificate – perhaps if a patient meets a weekly goal/attends a certain number of sessions in a week.

SECTION 4:

ACTIVITIES

This section gives a series of step by step guides for activities that you can lead, support or participate in with patients.

There are activity ideas to suit a variety of needs and leave access.

Ward games

These games are a great option for patients who may not want to formally participate in any ‘physical activity’ but might still be interested in activities.

All these games can be played with minimal equipment and can be played either on the ward or courtyard, so are good options for patients that may not be able to take leave.

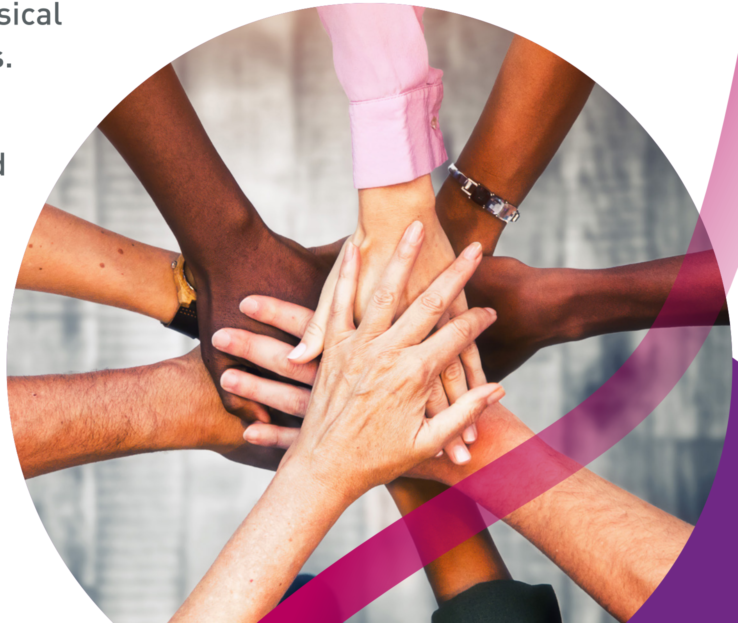

Paper cup skittles

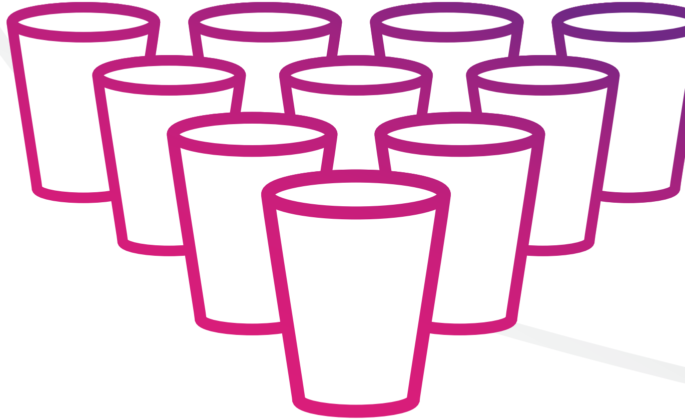

What you will need: 10 paper cups, foam ball.

How many people: 2-6.

How to do it: set up cups like the image. Each person has 2 attempts to see if they can knock down all the cups. You get a point for each skittle you knock down. You can play many rounds as you like, the winner is whoever has the most points at the end.

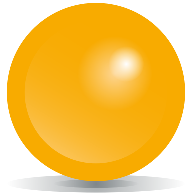

Cup-pong!

What you need: 10 cups and a ping pong ball, floor/table space.

How many people: 2, or 4 (to play as a team)

How to play: Arrange the cups in a triangle at the end of the floor/table.

Take turns to throw the ball into the other persons cup.

Keep playing until one person/team has no cups remaining. The team with no cups remaining loses, and the other wins.

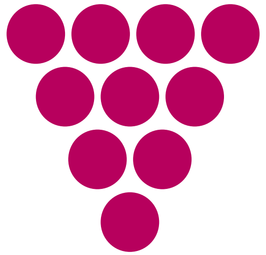

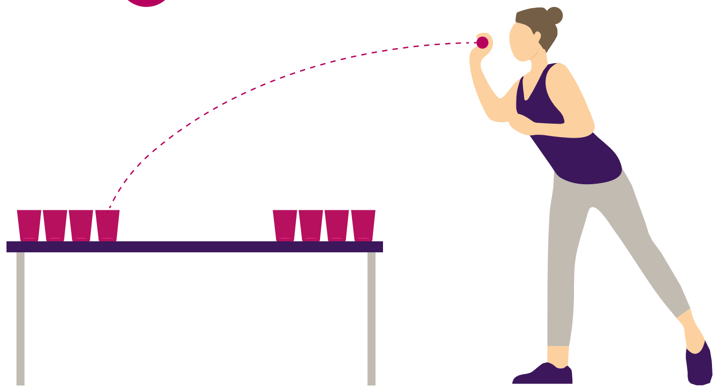

Gym workouts

This section gives a guide for some activities that you can do in the gym, using cardio equipment. All of these can be adapted based on patient needs.

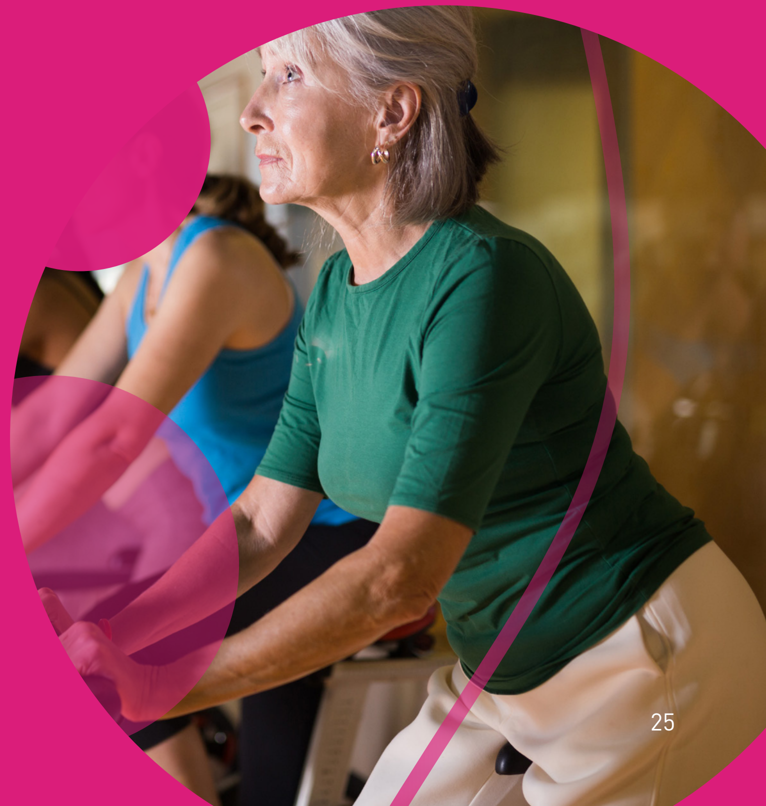

How to safely use cardio equipment in the gym

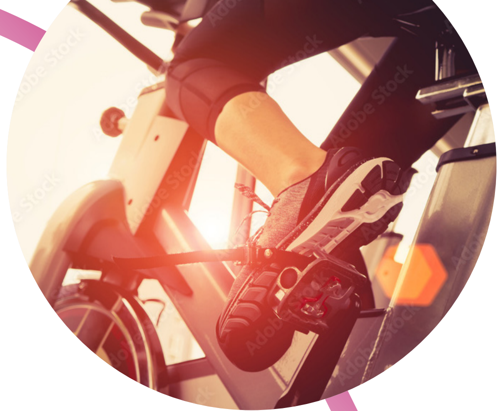

Cardio equipment are machines in the gym (typically treadmill, rower, stationary bike
and cross-trainer) which work your cardiovascular system.

Benefits of using cardiovascular equipment:

Can help to strengthen your heart, lungs and muscles.

Helps you to build stamina, endurance and overall fitness.

Dependent on the machine you use (not treadmill), using cardio equipment is low impact so can be beneficial for people who struggle with high impact physical activity.

Before you start:

Does the patient have any new health conditions that you need to be aware of?

Is the patient alert enough to be using a machine?

Any concerns? Check with the nurse in charge before using the gym.

How to use it: cross trainer

Cross-trainer: Get a good grip on the handlebars and then step onto the machine. The pedals may start to move initially when you step onto the 'feet' of the machine. Start slowly, without any resistance or incline. After a few minutes, you may want to increase the resistance. Remember to keep a slight bend in your knees throughout.

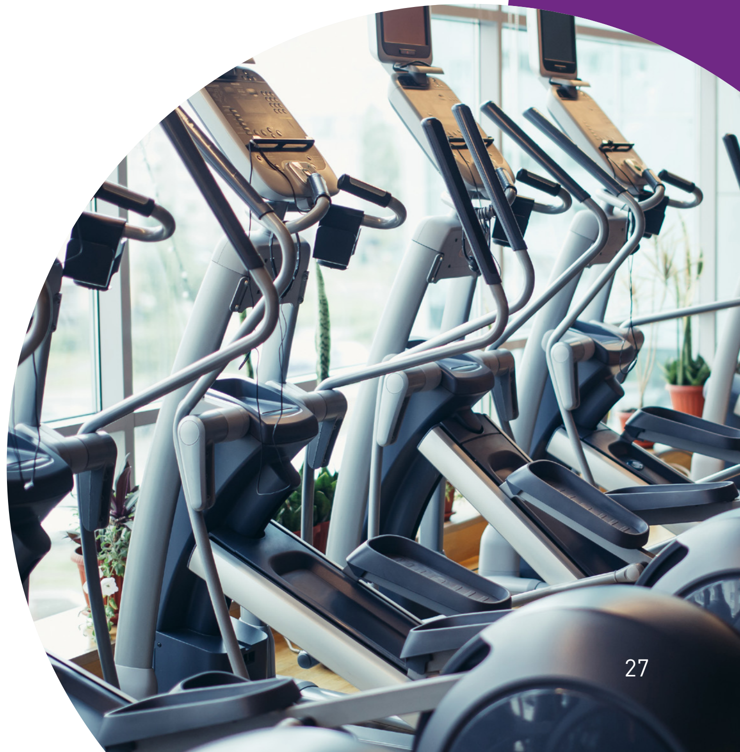

How to use it: Indoor cycle, also called a ‘spin bike’ or ‘stationary bike’

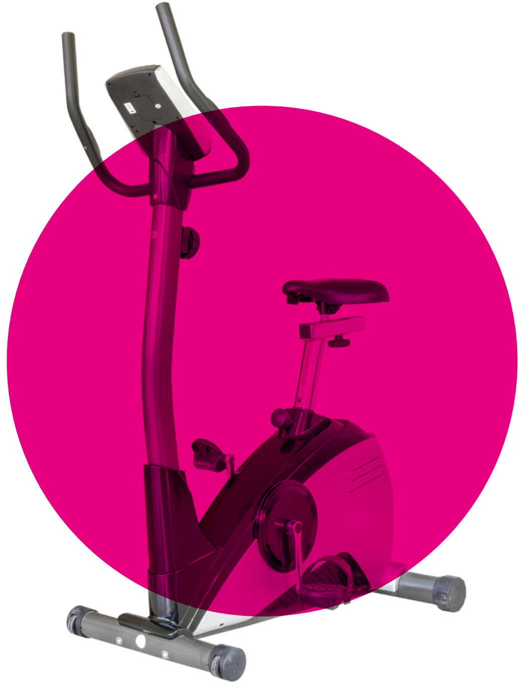

Before you start: Adjust your seat height. Ideally, when the pedal is at its lowest point you want your leg to be almost, but not entirely straight.

For your handlebars, you want to be able to reach for them comfortably. Then if there are straps on the pedals, strap your feet in. This will make cycling more comfortable.

How to use it: The treadmill

Before you start: familiarise yourself with the buttons on the machine. Mainly the start, stop and speed buttons.

Time to get moving: step onto the treadmill and press the ‘start’ button. This will make the treadmill move very slowly. You can gradually increase the speed if you feel able to depending on your fitness levels and experience. When your finished, press the stop button to slow the treadmill belt down.

Getting off: don’t jump off, once the treadmill belt has stopped you can use the rubber sides to help you get off.

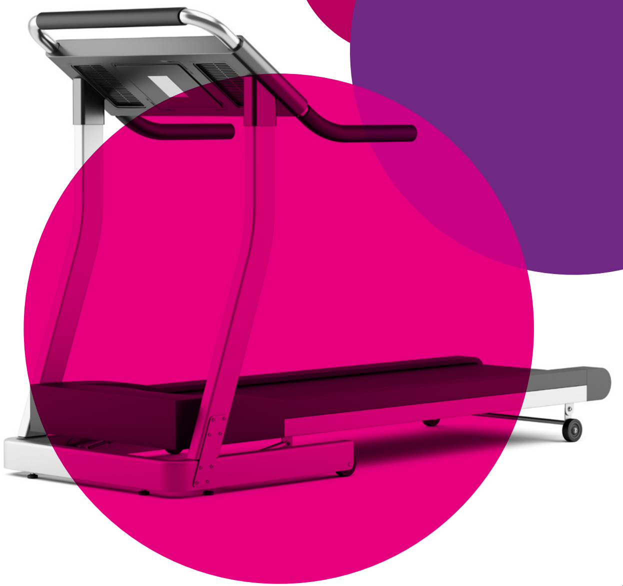

Cardio equipment workouts

These workouts can be done on any piece of cardio equipment in the gym (cross-trainer, treadmill, bike, rower).

1:1 workout	Pyramid workout	Interval Workout
Walk for 1 minute	Walk for 1 minute (rest 30 seconds)	15 seconds high intensity (45 seconds rest)
Jog/brisk walk for 1 minute.	Walk for 2 minutes (rest 30 seconds)	30 seconds high intensity
Repeat 3-5 times	Walk for 3 minutes (rest 30 seconds)	(30 seconds rest)
	Walk for 2 minutes (rest 30 seconds)	*High intensity means that you might be breathing heavily

Top Tips:

Put some music on

Do the workout together (with another patient or member of staff) – this can make it much more enjoyable for both staff and patients

All of these workouts can be adapted to be done outside if you have leave – maybe by walking, jogging or using the green-gym.

Individual activities on the wards

This section gives examples of activities that can be conducted individually, or in a small group on the ward. These may be useful for patients who might be interested in some structured physical activity but may want to be flexible in the duration and type of physical activity they do.

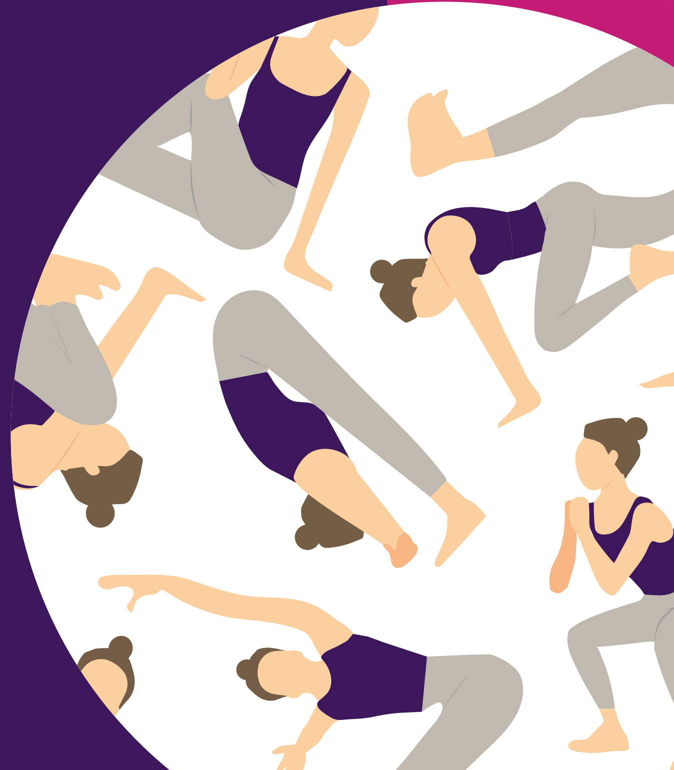

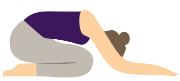

1. Child's pose
Deepen the breath.
Keep your hands far forward and elbows off the ground, stretching the upper body

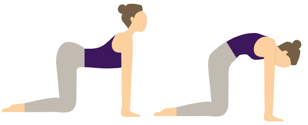

2. Cat-Cow stretch
Inhale - drop the belly, cow pose.
Exhale - round the spine, cat pose.
Repeat 5-8 times

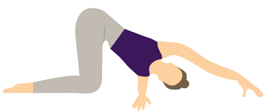

3. Threat the needle
Stay for 3-5 breaths on each side

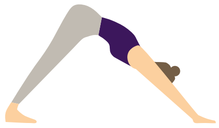

4. Downward facing dog
Bend knees one by one, stretching out the back of the legs

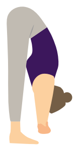

5. Ragdoll
Hug elbows, knees can be bent, releae neck and spine.
Rock side to side

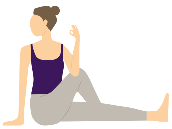

5. Seated spinal twist
Stay for 3-5 breths on each side

5-minute yoga sequence

This 5-minute yoga sequence is a great option for some low intensity activity that can be done anywhere.

It is good to focus on breathing throughout this sequence as this can help with relaxation and maintaining posture.

Want to do some more yoga?

Scan this QR code for a 20-minute beginner's video

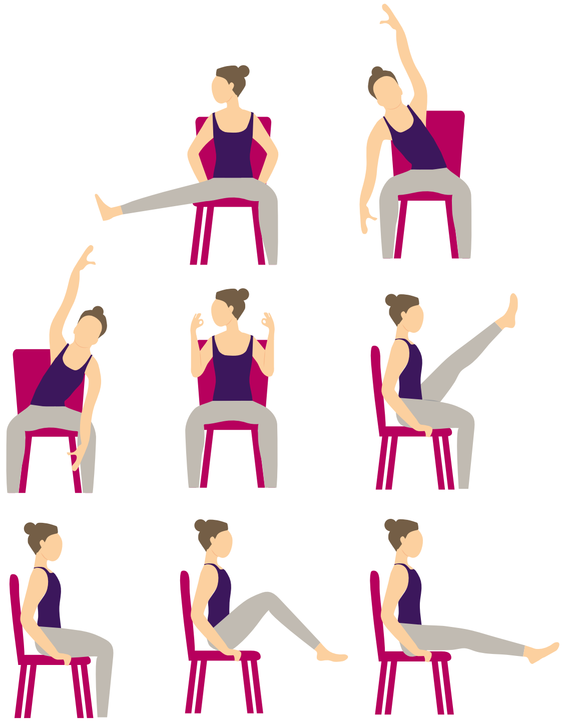

Chair based yoga exercises

Here are some simple yoga exercises that you can do from your chair.

You can work your way through this sequence as many times as you like.

Focus on taking slow deep breaths and only do movements that are comfortable for you.

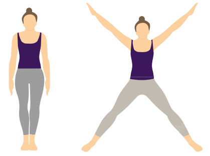

1. Jumping Jacks
3 sets / 45 seconds

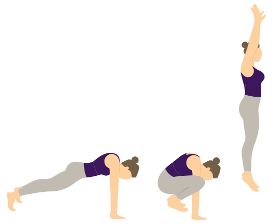

2. Burpees
3 sets / 45 seconds

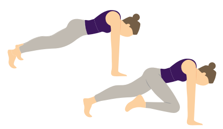

3. Mountain climbers
3 sets / 45 seconds

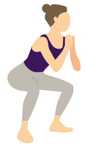

4. Bodyweight squat
3 sets / 45 seconds

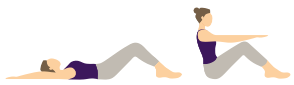

5. Sit ups
3 sets / 45 seconds

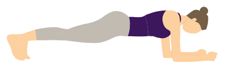

5. Plank
3 sets / 45 seconds

5-minute circuit

This is a great option for patients who might want to exercise at a higher intensity.

The picture gives an indication of some of the physical activity that you can include. However, you can give the patient the option to choose others that they might like.

The lengths of the intervals and rest periods will vary dependent on fitness levels so let patients dictate what might be most suitable for them.

Check out this QR code for some more low impact circuit ideas.

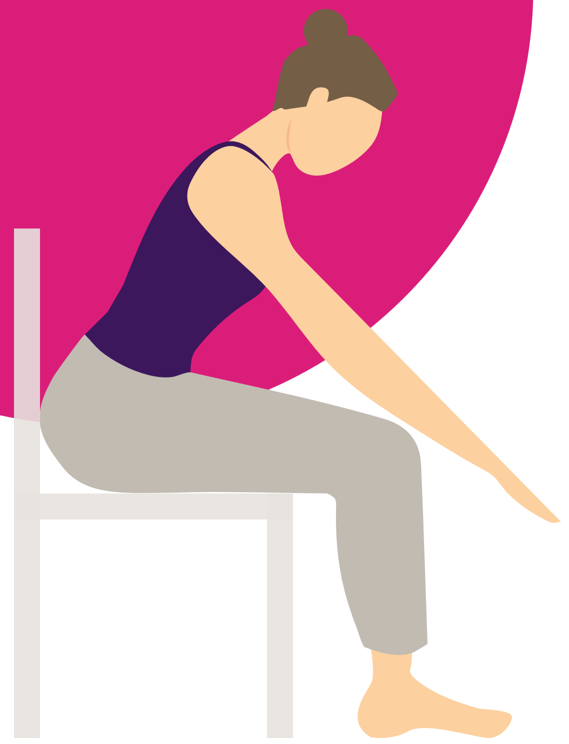

10-minute chair-based workout

Check out this link for a video by Joe Wicks on chair-based circuits.

Games to play in the courtyard

This section gives examples of activities that can be played in small grounds in the courtyard or outdoor space

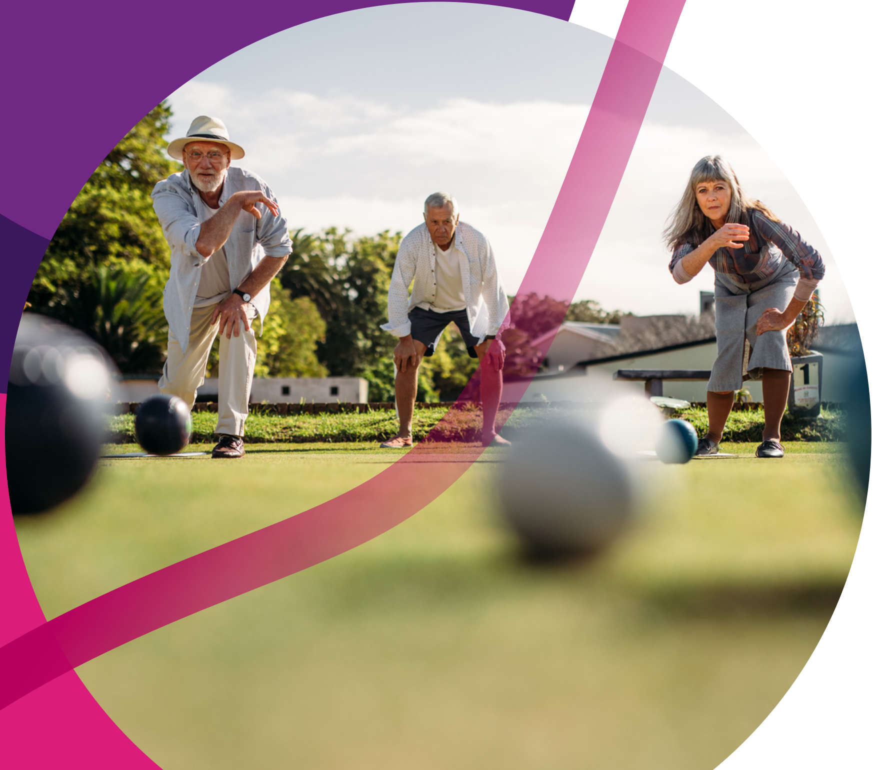

Shadow tag!

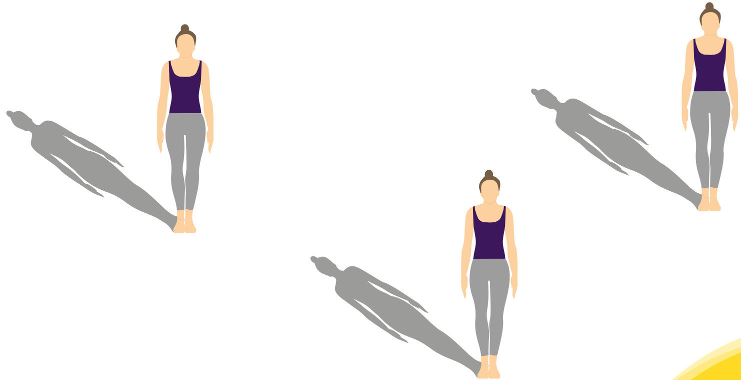

This is a great option for a sunny day and doesn't require any equipment

What you will need: yourself!

How many people can play: 3+

How to play: the aim of the game is to tag each others shadows. Once a persons shadow is tagged, they are out. The winner is the last person left.

H-O-R-S-E

H-O-R-S-E is a game that you can play in the courtyard/sports hall if you have basketball hoop and a ball.

What you will need: yourself!

How many people can play: 2+

How to play: The first player takes a shot at the basket, followed by the second player.

If a player fails a shot, they get the letter H. Each time someone fails a shot, they get another letter, eventually spelling out the word HORSE.

Whoever spells out HORSE first loses.

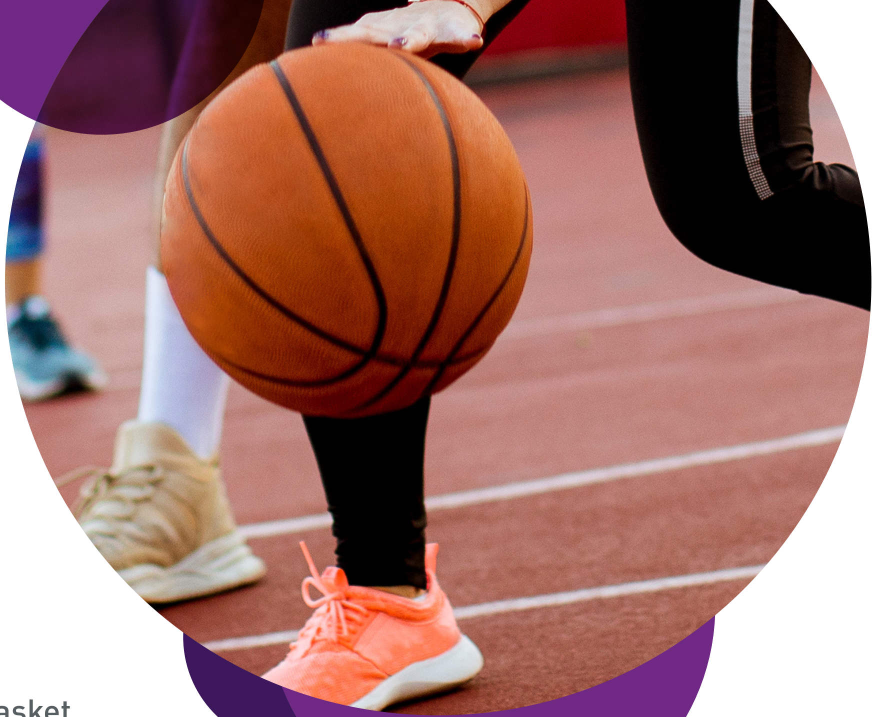

Got chalk? These are some great options for games in the courtyard with some chalk!

Chalk Bullseye

Use various colours of chalk to draw concentric circles on a wall or floor.

Within each circle write points values (e.g. 10, 20,30)

Throw an item onto the bullseye – the first person to get 100 points wins!

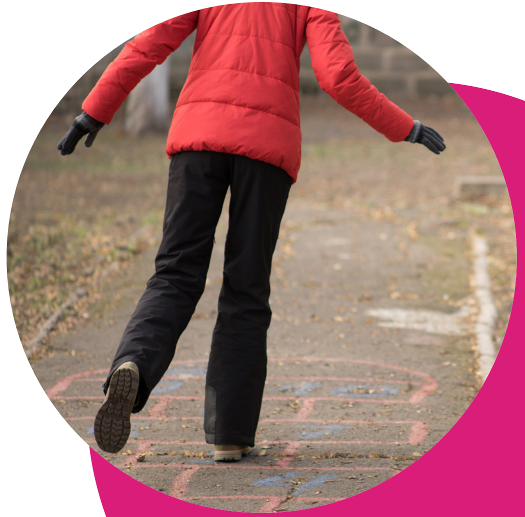

Scan this QR code for more games that can be played with chalk.

Activities for the grounds

This section gives examples of activities that can be done in the grounds

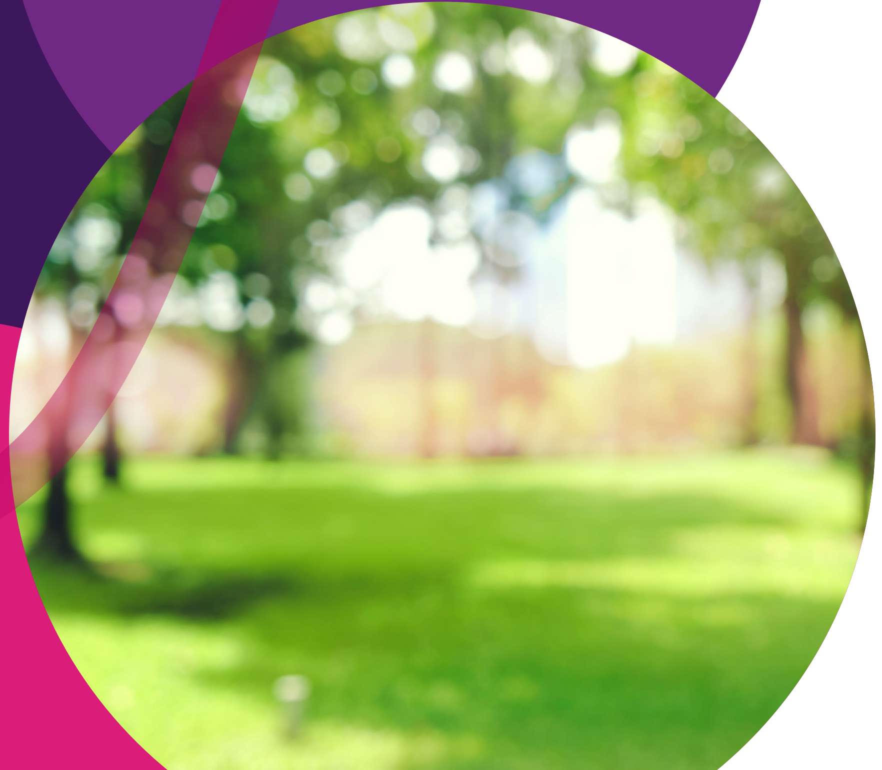

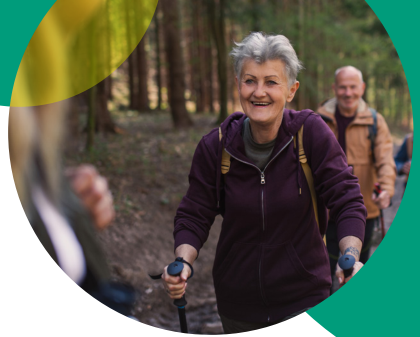

Nature walks

When walking in the grounds, see how many things you can spot.

	Record how many you spot here		Record how many you spot here
Dandelions		Butterflies	
Ladybirds		Robin	
Oak Tree		Bee	
Daisy		Pine Cone	

Green gym

You should ask the patient how they would like to engage with the green gym – they may have ideas of what they might like to do. Below, here are some ideas to get you started.

Getting started with the green gym:

Start with 3-5 minutes easy exercise on a cardio machine.

Choose a weights machine and do 3x10 repetitions (you can do more or less depending on preferences).

Do a 3-5 minute warm down on a cardio machine (or walk back to the ward).

Being active in green space has been shown to reduce stress and improve mental wellbeing.

Scan this QR code for guidance on how to use green-gym equipment

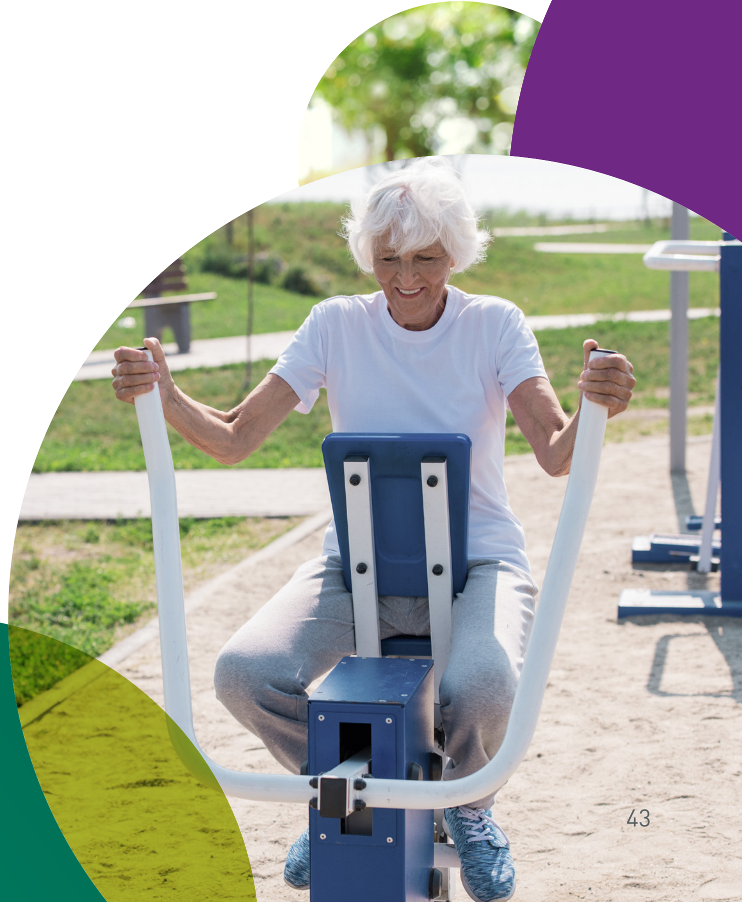

SECTION 5:
RESOURCES

This section provides several resources that may be helpful to support patients in physical activity. These can be photocopied or printed directly from our online resources.

CERTIFICATES

PHYSICAL ACTIVITY PLANNER

PHYSICAL ACTIVITY MOOD RECORDER

GOAL SETTING

STAR CHART

My move chart

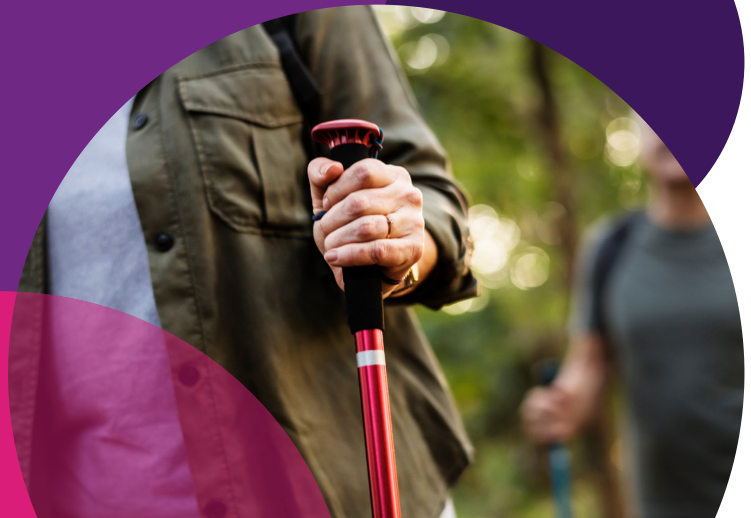

My goal this week is to:

Day	What I did	How long I did it for	How did I feel?	Mood before	Mood after	Comments
Monday						
Tuesday						
Wednesday						
Thursday						
Friday						
Saturday						
Sunday						

BRONZE

Bronze award is presented to

....

For:

....

Signed: .... Date: ....

SILVER

Silver award is presented to

....

For:

....

Signed: .... Date:

GOLD

Gold award is presented to

....

For:

....

Signed: .... Date: ....

My star chart

I earn stars for:

					REWARD! ☆
					REWARD! ☆
					REWARD! ☆

When I earn 5 stars I earn:

Scan these QR codes for links to printable resources

Goal setting chart

Star Chart

Bronze Certificate

Silver Certificate

Gold certificate
