# Supplementary material for: The role of public and patient involvement in designing a web-based, physical activity application for individuals with severe mental illness
Source: Res Involv Engagem. 2025 Jul 21;11:86. doi: 10.1186/s40900-025-00735-x (PMC12278672; doi:10.1186/s40900-025-00735-x)
Supplement: Supplementary file 4 — Supplementary Material 4 [file 40900_2025_735_MOESM4_ESM.pdf]

PPI MEETING 1 AGENDA

Time:

Date:

Location:

Meeting Presenter:

Attendees:

01. Introduction

- Researcher introduction, explain the research focus and objectives and the importance of PPI.
- Everyone to introduce each other and share a fun fact of each other.
- Answer any questions.

02. PA experiences and the Environment

- Barriers and facilitators within your own setting to participating in PA
- Present supports for PA- your own setting, staff, environment. What helps you do PA right now? Facilities?
- PA you enjoy/ don't enjoy.
- What does PA do for you?
- Environment in supporting you do PA.

03. Digital Health Knowledge

- Do you own any digital tools?
- If so, have you used apps?
- Do you know what an app is?
- What would stop you from using a PA app?
- Need to be shown digital usage? Or comfortable?

04. Methodology Discussion

- Explanation of the intervention- 10 week intervention (phase 1), 6 weeks intervention (phase 2) and follow up (+3 months).
- GENEActiv shown.

05. Any other business and closing.

- Thank all for taking their time and coming
- Answer any questions.
- Decide on next date and location of meeting, suitable to all.

TemplateLAB

PPI MEETING 2 AGENDA

Time:	Meeting Presenter:
Date:	
Location:	Attendees:

01. Introduction

- Recap of last meeting

02. Physical Move More Toolkit

- Move More Toolkit shown.
- Throughout, perceptions, barriers of the toolkit?
- To go through all games- keep, remove, or alter?
- What facilities are present in your setting which may be added here?
- Trial out games- Activities made fun.
- Decide on games within the toolkit.

03. Toolkit as an app

- Toolkit display on app- font size, font, color scheme, layout.
- Visuals?
- Quotes?
- Video instructions?
- QR codes incorporated around the hose?
- View other apps of similar layouts.

04. Any other business and closing.

- Thank all for time.
- Answer questions.
- One for all vouchers.

TemplateLAB
