# Supplementary material for: The role of public and patient involvement in designing a web-based, physical activity application for individuals with severe mental illness
Source: Res Involv Engagem. 2025 Jul 21;11:86. doi: 10.1186/s40900-025-00735-x (PMC12278672; doi:10.1186/s40900-025-00735-x)
Supplement: Supplementary file 5 — Supplementary Material 5 [file 40900_2025_735_MOESM5_ESM.docx]

**Location**

**Friends**

**Physical Activity options**

**Knowledge about physical activity**

**My mental health**

**Lack of confidence**

**Lack of equipment**

**Facilities**

**Staff**

**Environment**

**Space**

**Equipment**

**Time**

**Beliefs**

**Concerned they could injure**

**Don’t know what to do**

**Motivation**

**Medication**

**Stigma**

**Flexibility**

**Choice**

**Better mood**

**Social support**

**Better health**

**Feeling better**

**No digital tool access**

**Difficult to understand**

**What helps you do physical activity right now?**

**
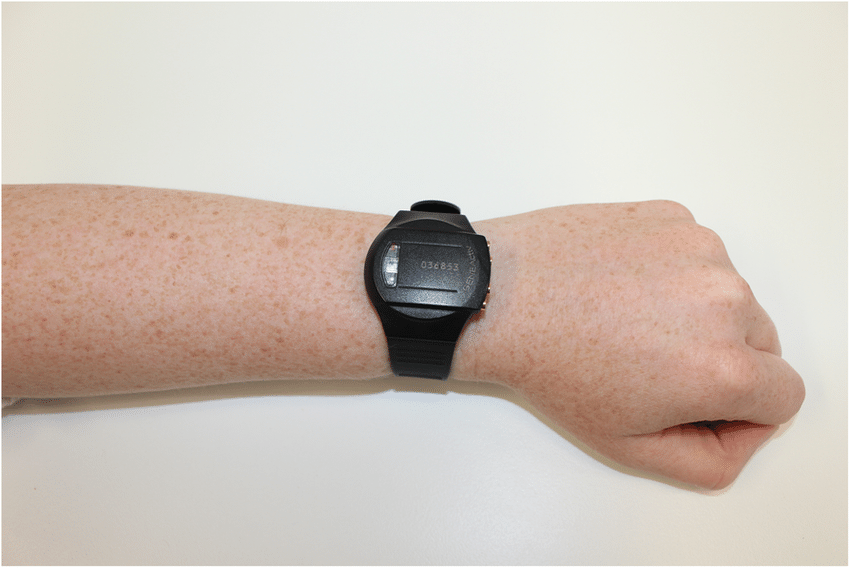

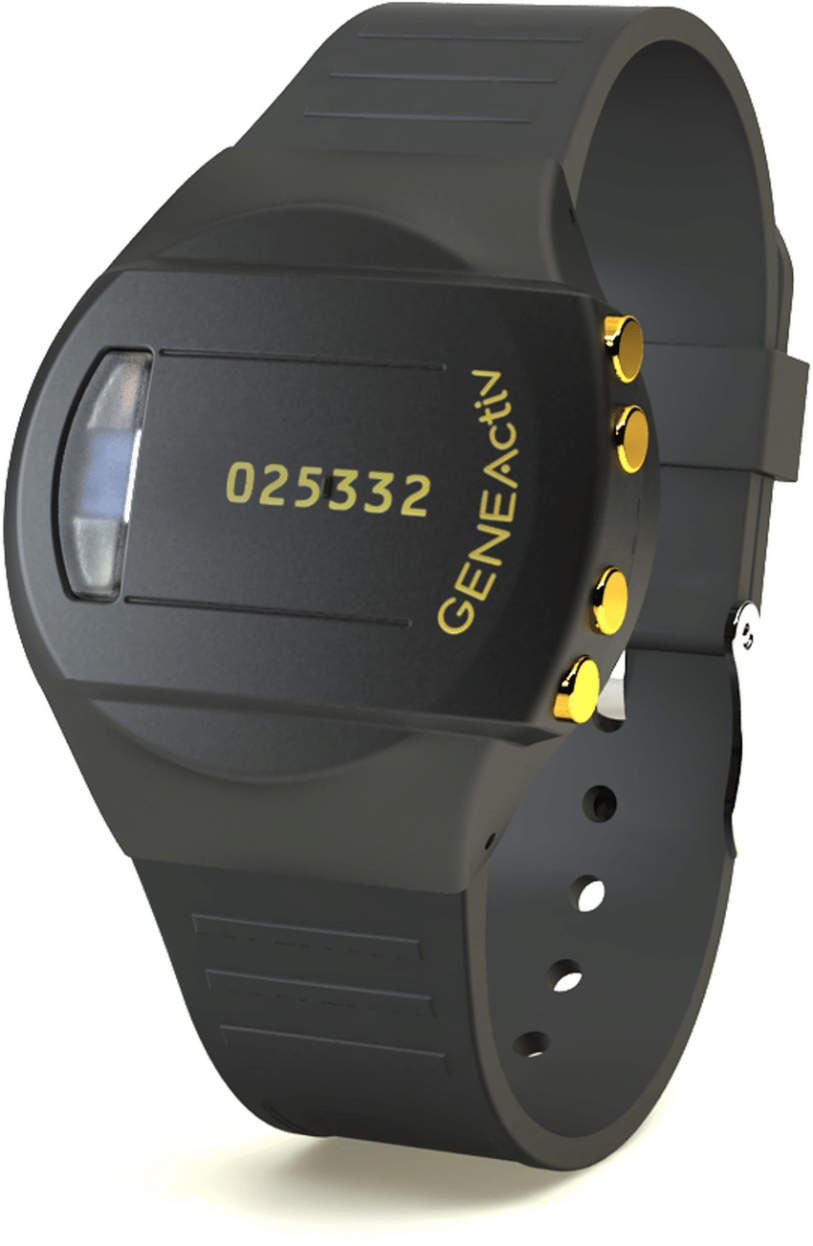
**

**QR code**

**
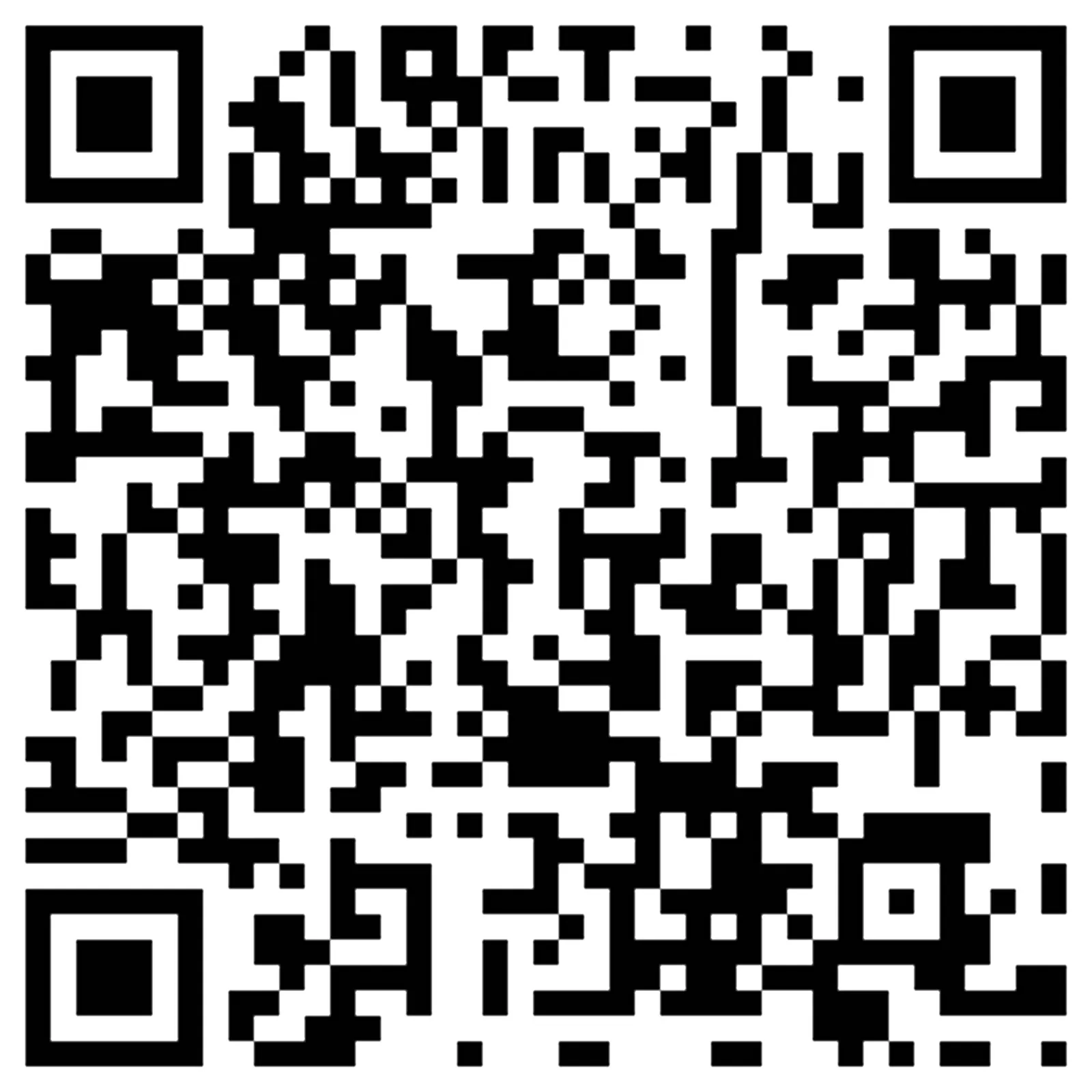
**

**App layout**

**
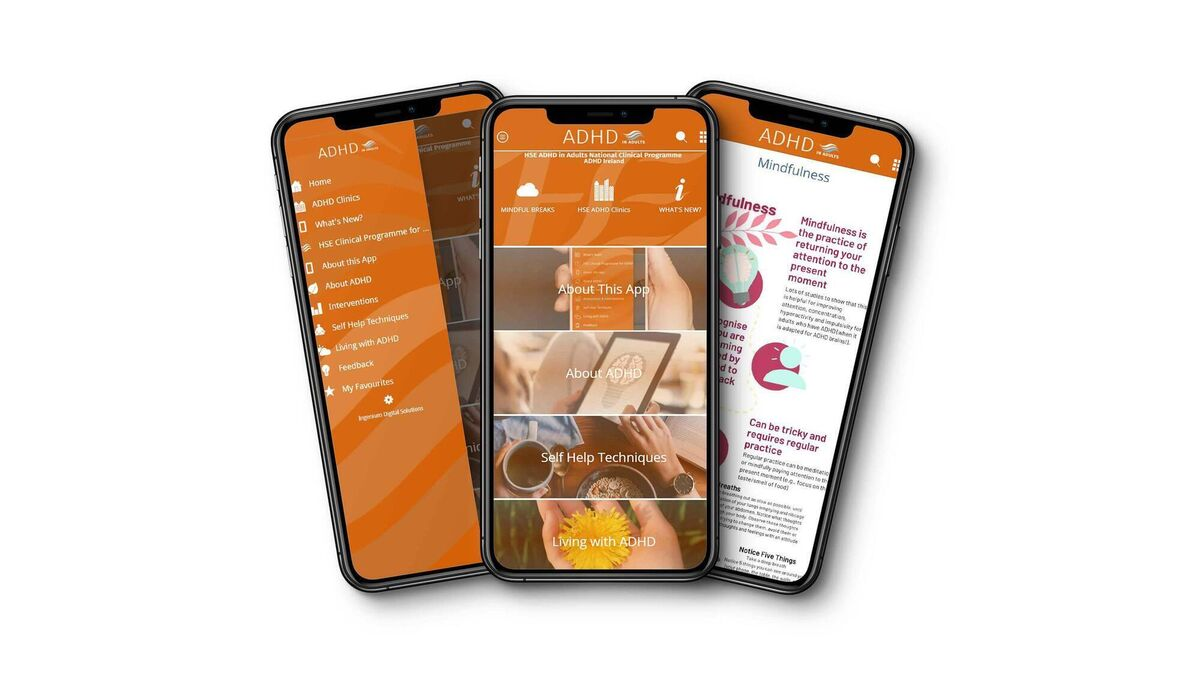
**

**
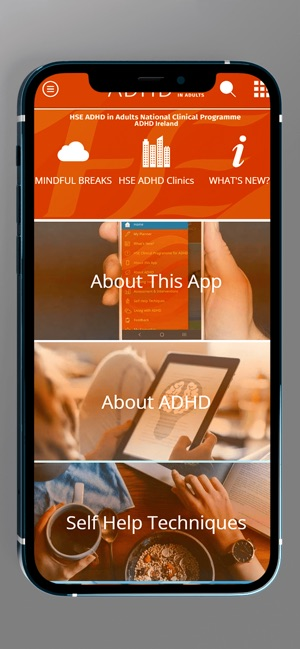

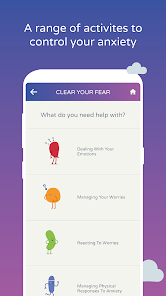

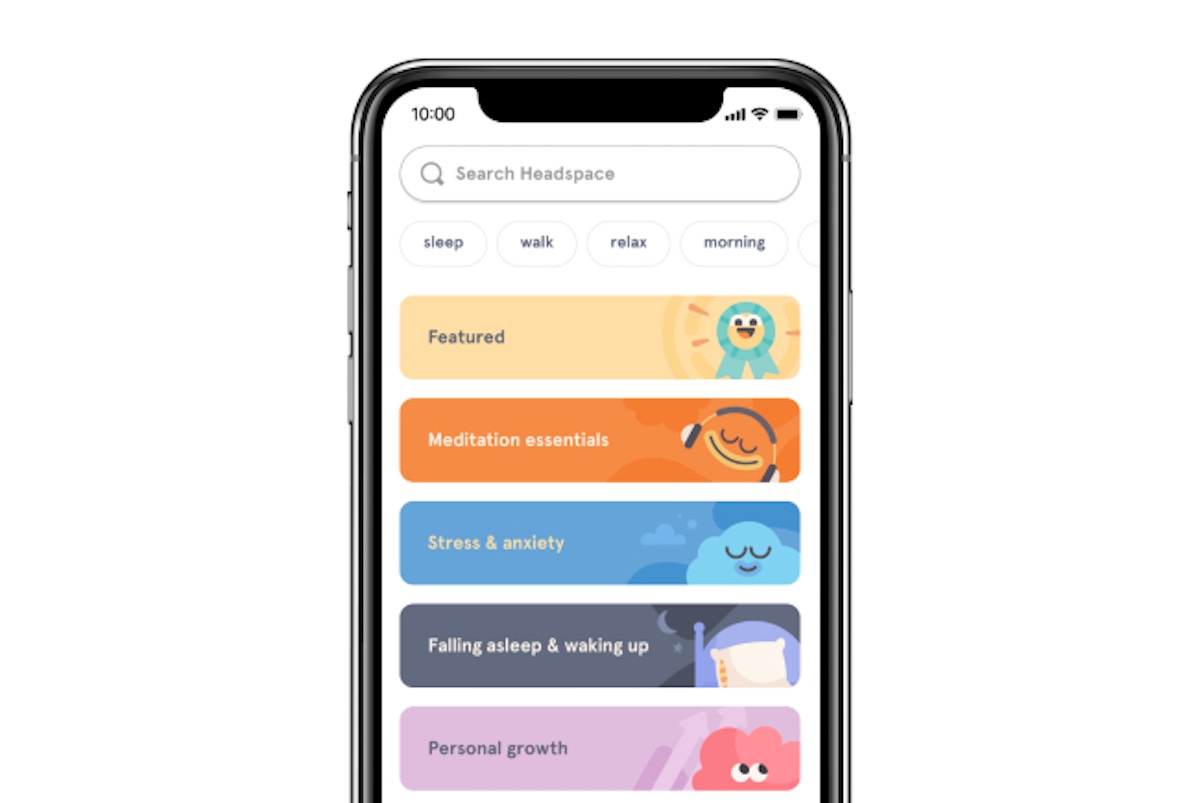
**
