# Supplementary figures and images for: The role of public and patient involvement in designing a web-based, physical activity application for individuals with severe mental illness
Source: Res Involv Engagem. 2025 Jul 21;11:86. doi: 10.1186/s40900-025-00735-x (PMC12278672; doi:10.1186/s40900-025-00735-x)

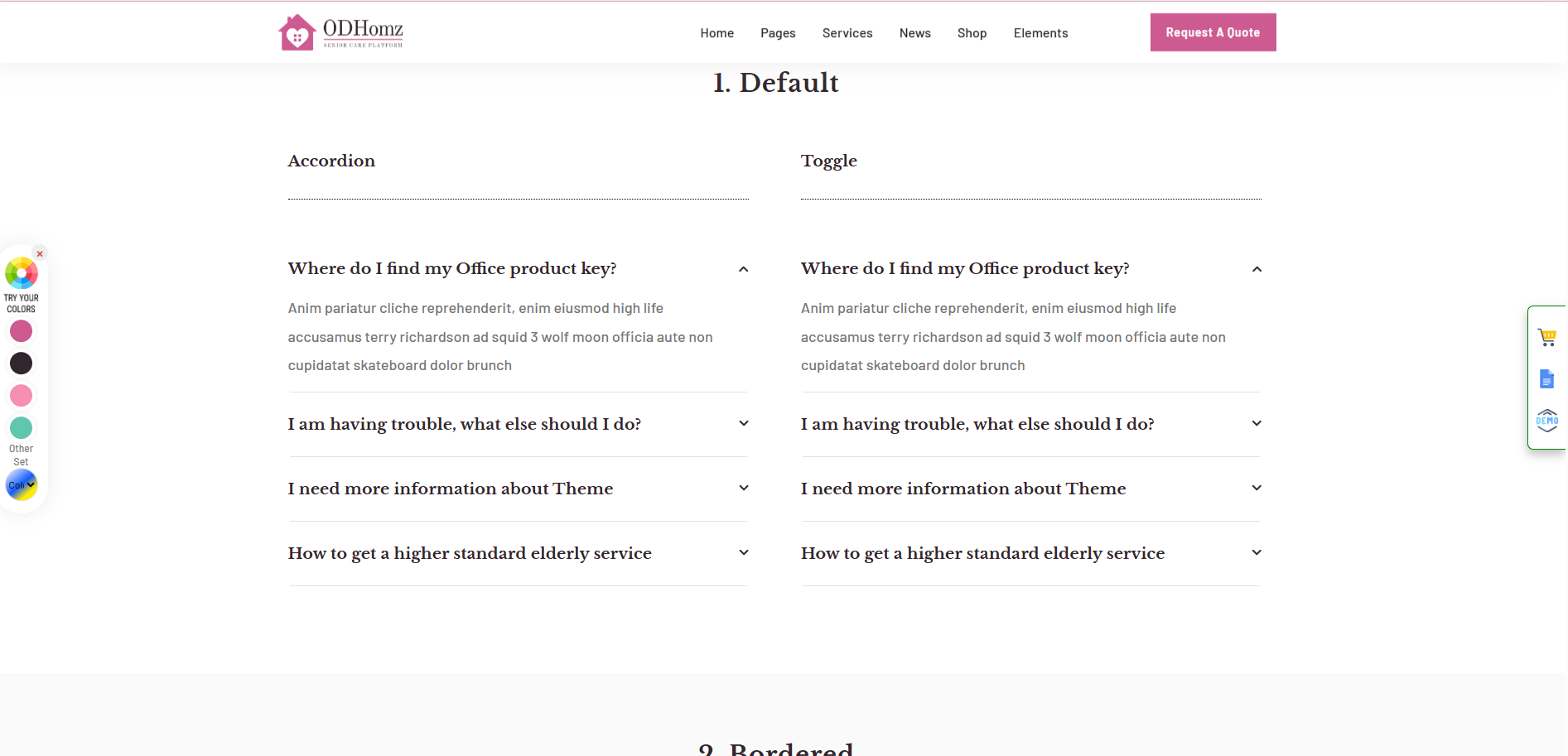

Supplement: Supplementary file 6 — Supplementary Material 6 [file 40900_2025_735_MOESM6_ESM.png]

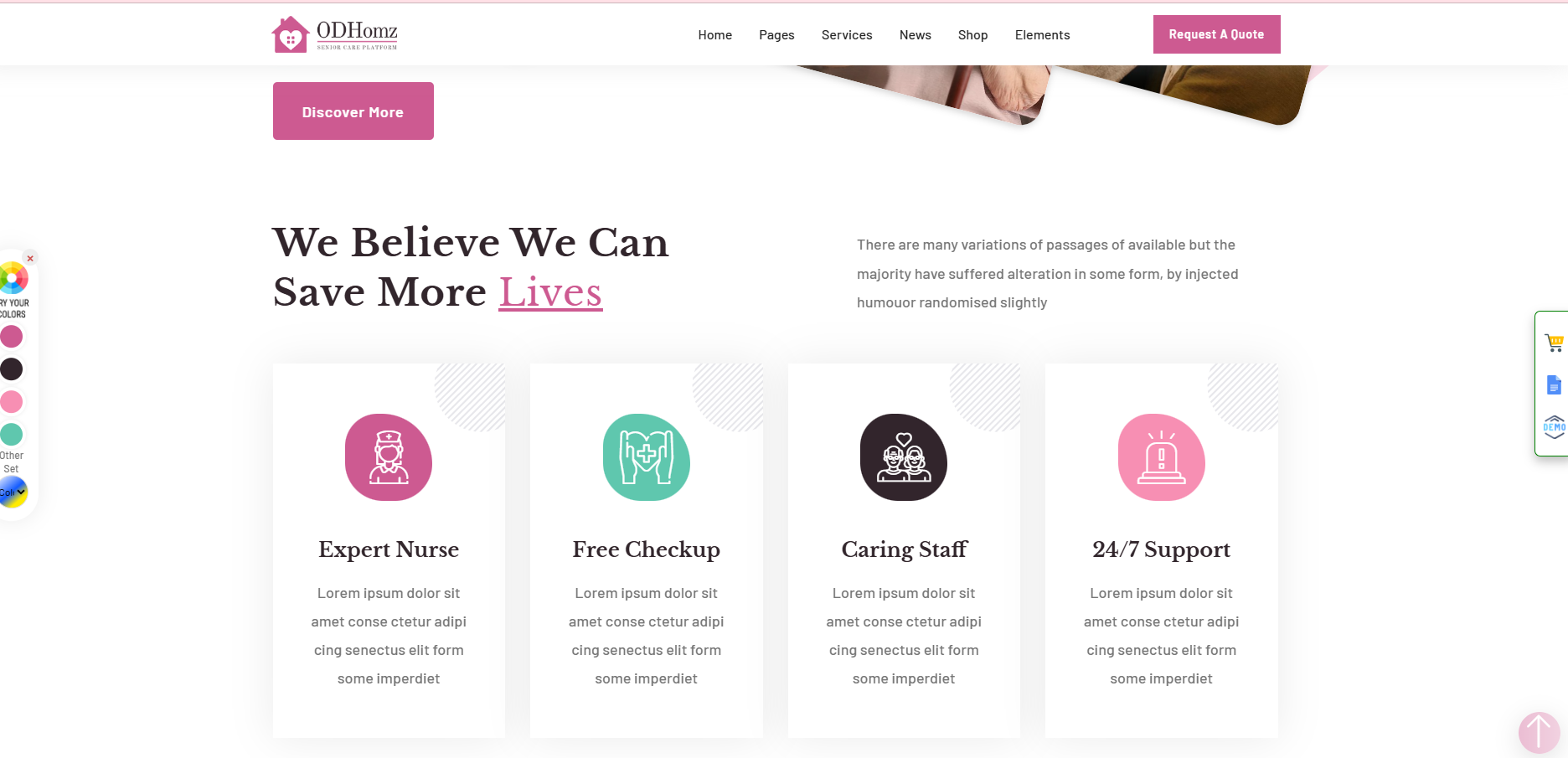

Supplement: Supplementary file 7 — Supplementary Material 7 [file 40900_2025_735_MOESM7_ESM.png]

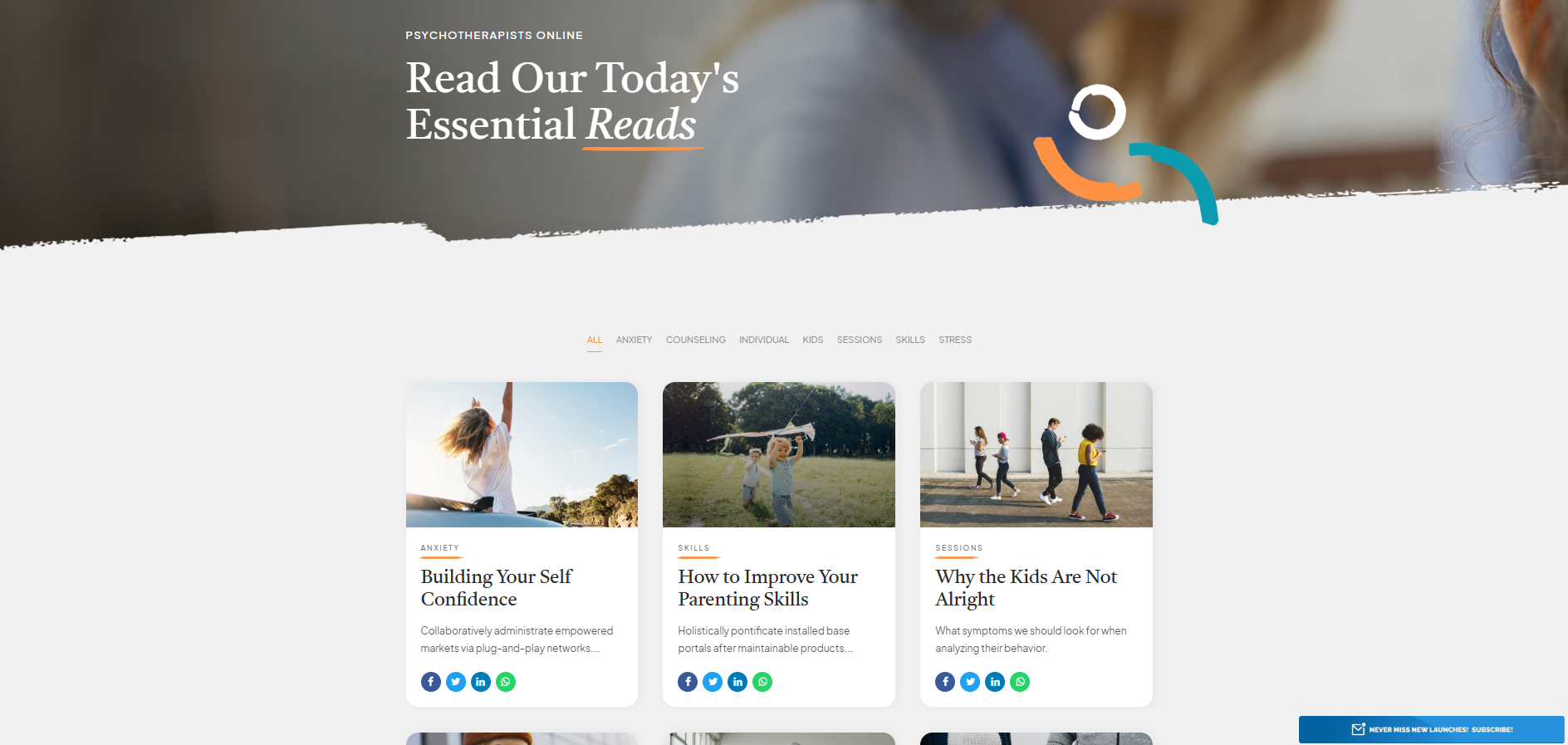

Supplement: Supplementary file 8 — Supplementary Material 8 [file 40900_2025_735_MOESM8_ESM.png]

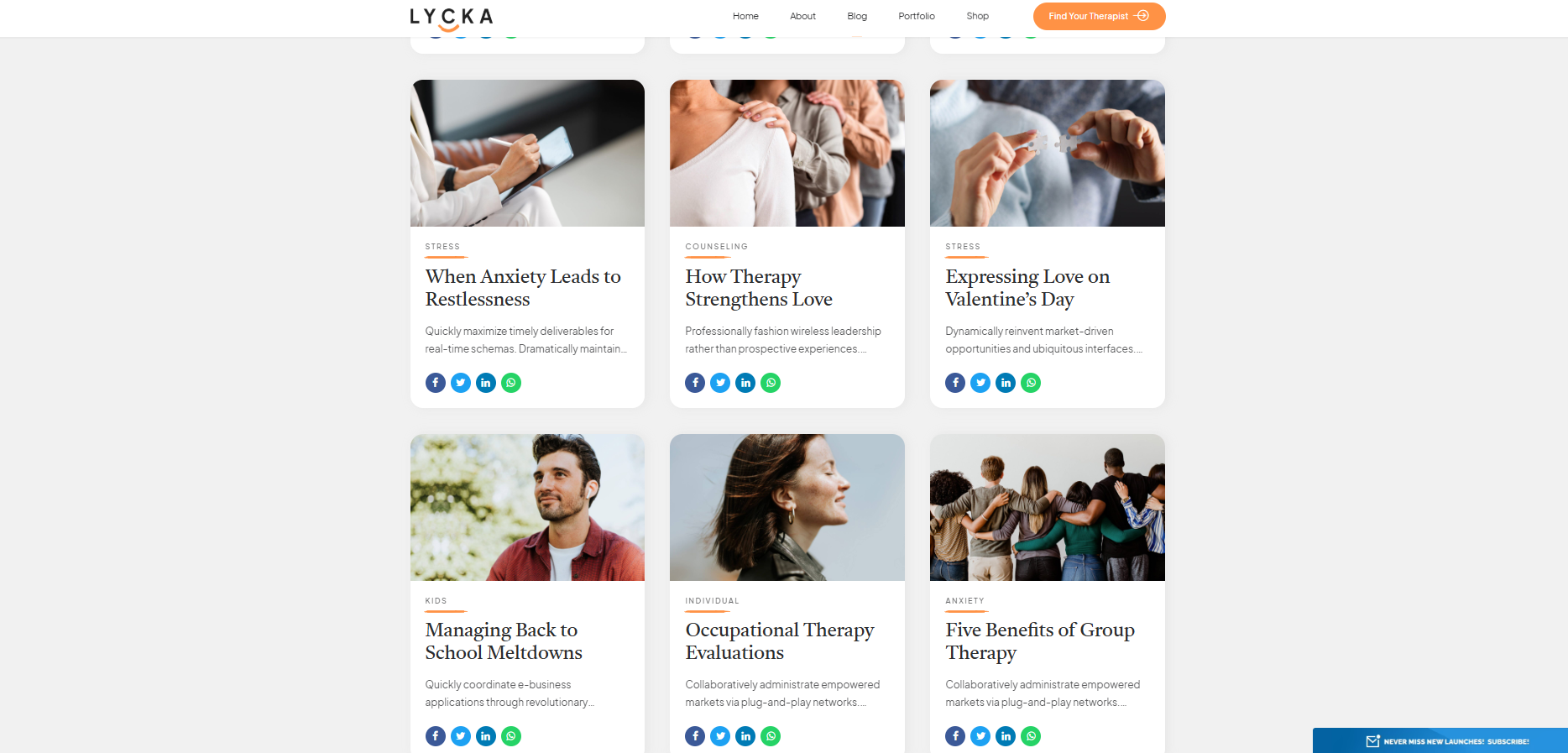

Supplement: Supplementary file 9 — Supplementary Material 9 [file 40900_2025_735_MOESM9_ESM.png]
