# Supplementary material for: The role of public and patient involvement in designing a web-based, physical activity application for individuals with severe mental illness
Source: Res Involv Engagem. 2025 Jul 21;11:86. doi: 10.1186/s40900-025-00735-x (PMC12278672; doi:10.1186/s40900-025-00735-x)
Supplement: Supplementary file 10 — Supplementary Material 10 [file 40900_2025_735_MOESM10_ESM.docx]

**Supplementary File**

*GRIPP2- SF*

| Section and topic | Item | Reported on page No |
| --- | --- | --- |
| 1: Aim | Report the aim of PPI in the study | 7 |
| 2: Methods | Provide a clear description of the methods used for PPI in the study | 8-12 |
| 3: Study results | Outcomes—Report the results of PPI in the study, including both positive and negative outcomes | 12-21 |
| 4: Discussion and conclusions | Outcomes—Comment on the extent to which PPI influenced the study overall. Describe positive and negative effects | 21-28 |
| 5: Reflections/critical perspective | Comment critically on the study, reflecting on the things that went well and those that did not, so others can learn from this experience | 21-28 |
