# Supplementary material for: The role of public and patient involvement in designing a web-based, physical activity application for individuals with severe mental illness
Source: Res Involv Engagem. 2025 Jul 21;11:86. doi: 10.1186/s40900-025-00735-x (PMC12278672; doi:10.1186/s40900-025-00735-x)
Supplement: Supplementary file 11 — Supplementary Material 11 [file 40900_2025_735_MOESM11_ESM.pdf]

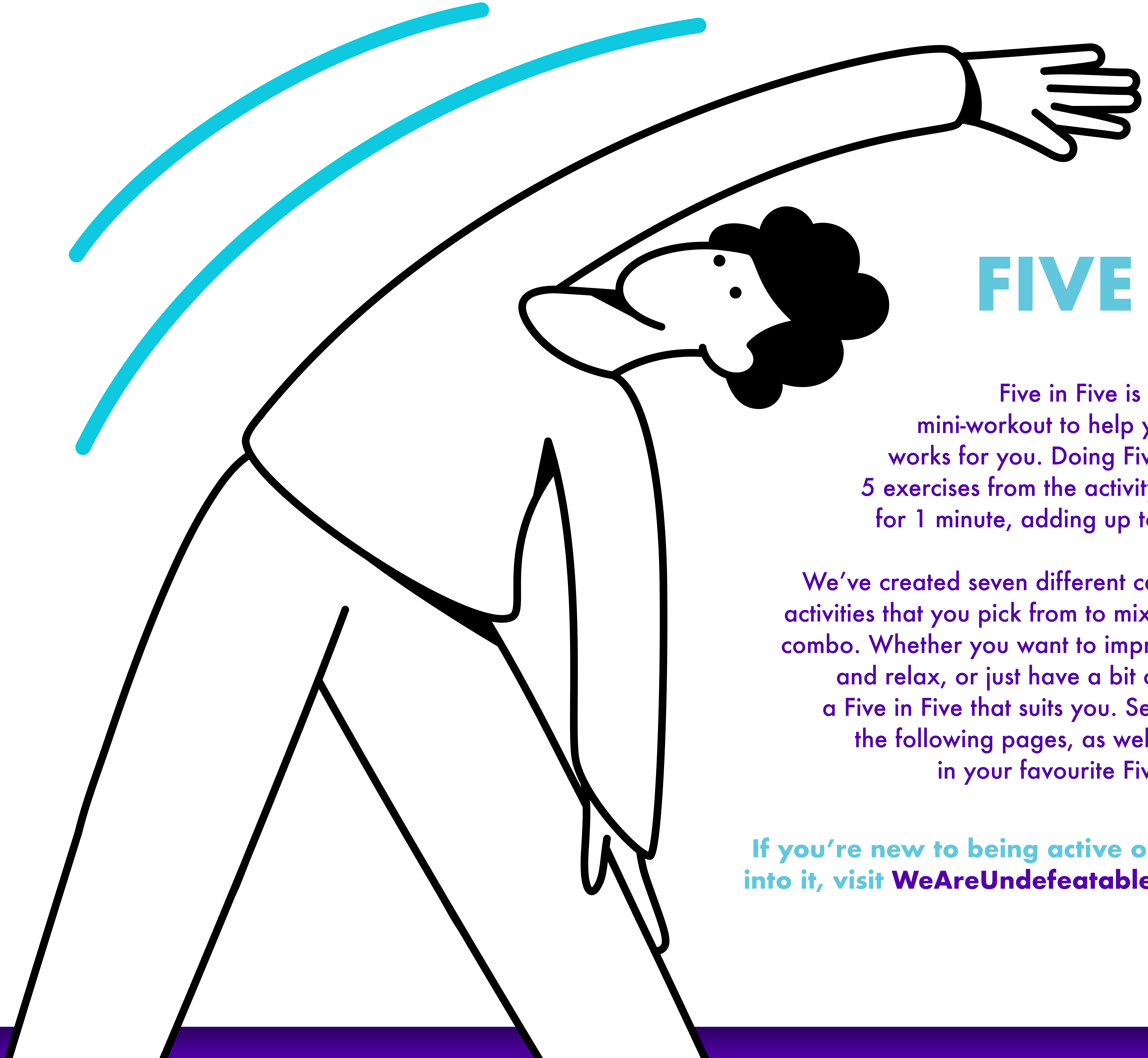

WE ARE UNDEFEATABLE

FIVE IN FIVE

Five in Five is a completely customisable mini-workout to help you move in whatever way works for you. Doing Five in Five is simple: choose 5 exercises from the activity library and do each one for 1 minute, adding up to a 5 minute mini-workout.

We've created seven different categories, each with a few activities that you pick from to mix and match your favourite combo. Whether you want to improve your strength, unwind and relax, or just have a bit of fun, there's bound to be a Five in Five that suits you. See some activity options on the following pages, as well as space for you to write in your favourite Five in Five on the last page.

If you're new to being active or are just getting back into it, visit WeAreUndefeatable.co.uk/getting-started

WARMING UP

Activities to get your body warmed up that require no equipment.

Doing Five in Five is simple:
Choose 5 exercises from the activity library and do each one for 1 minute, adding up to a 5 minute mini-workout.

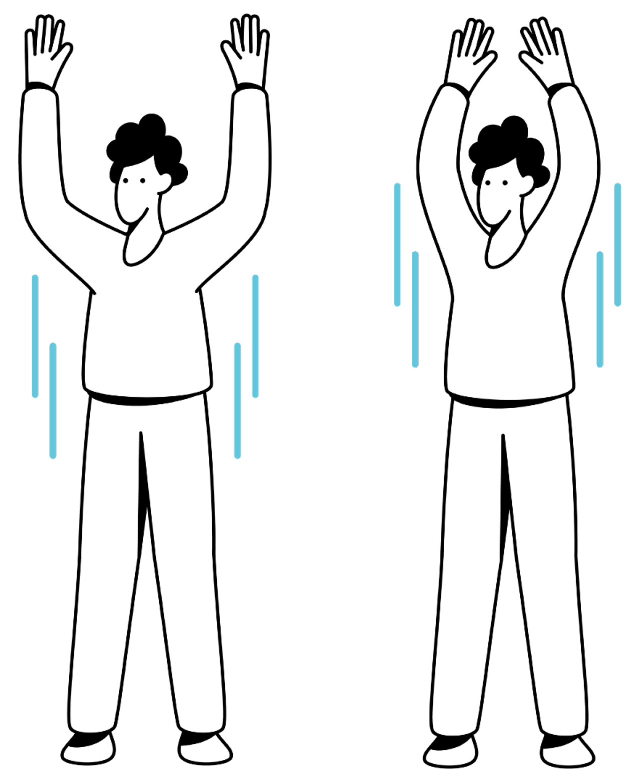

Push and Lift

Time to lift! Start with your hands in front of your shoulders and then push up overhead.

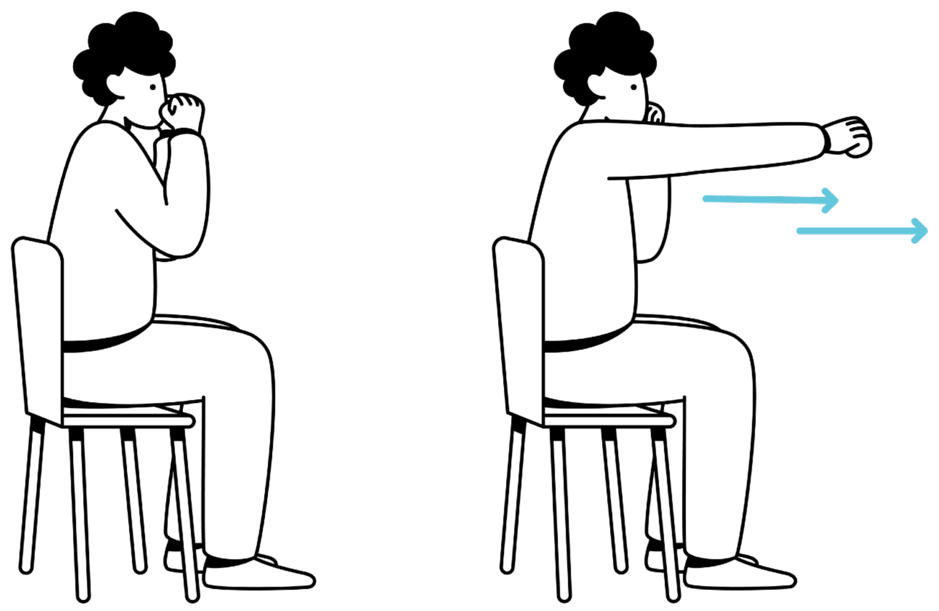

Seated Punches

While seated or standing, reach your arms out to hit an imaginary punching bag!

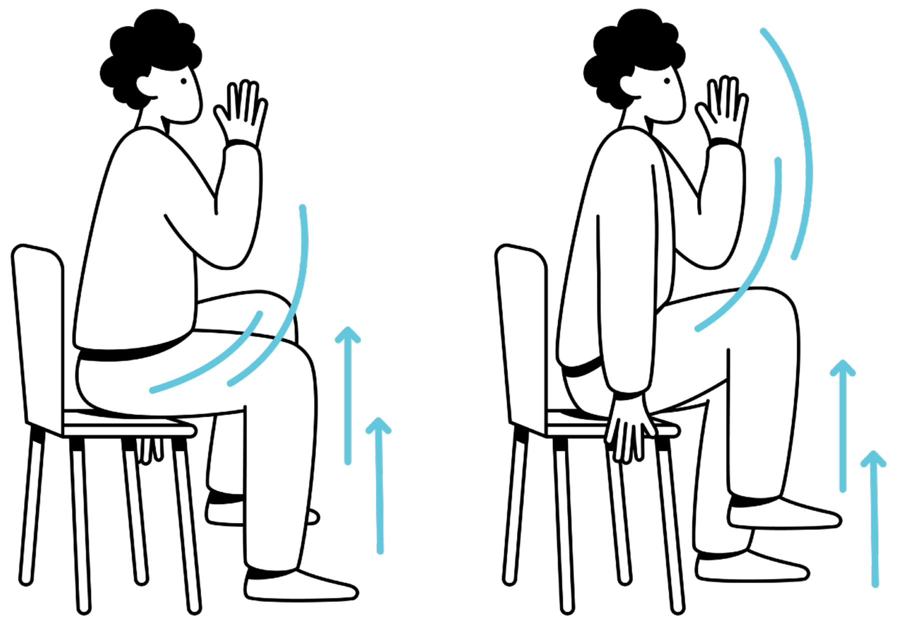

Seated March

Walk on the spot and gradually increase arm swings and knee lifts until you're marching. If you prefer to stand, you can march on the spot.

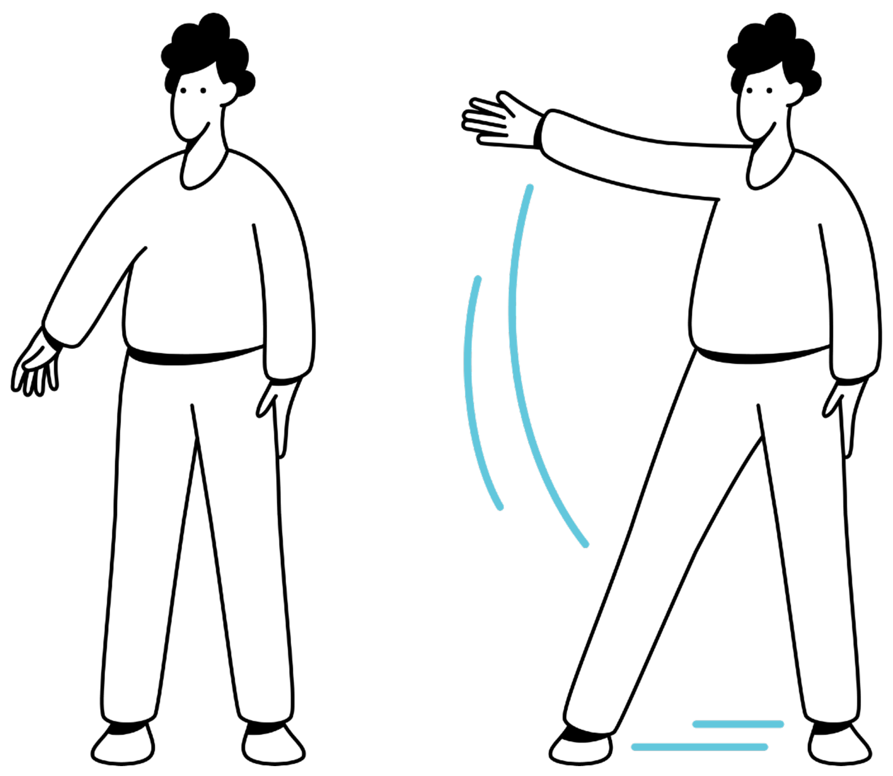

Half Jacks

Step to the side and lift one arm on your right side, then your left. If you're seated, you can do overhead claps instead!

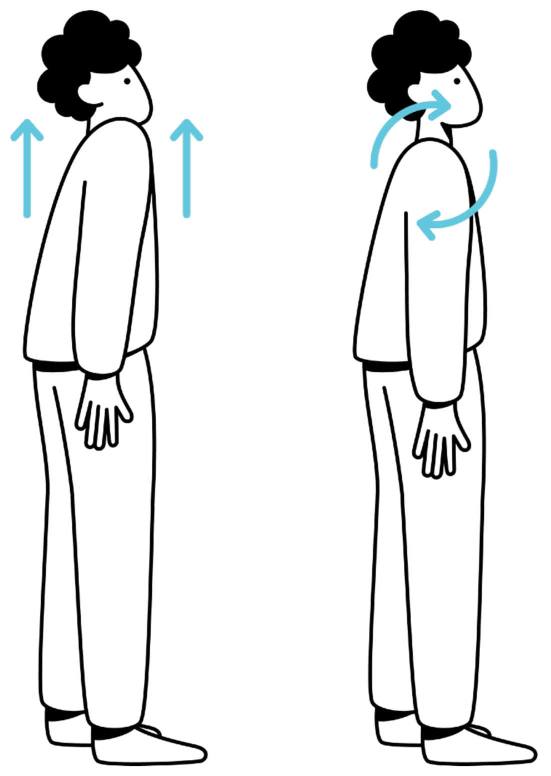

Shoulder Roll

Roll your shoulders around, first forwards, then backwards. After a little while, raise your arms for an arm roll, too!

STRESS-BUSTING

Stretching and other activities to unwind.

Doing Five in Five is simple:
Choose 5 exercises from the activity library and do each one for 1 minute, adding up to a 5 minute mini-workout.

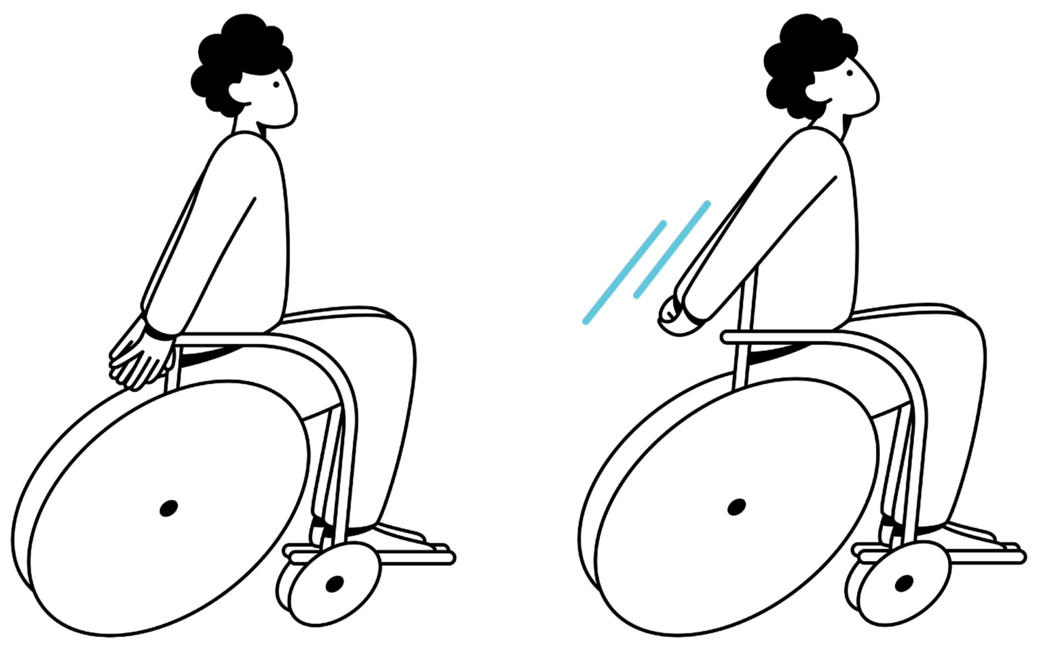

Stretch With Interlocked Hands

Interlock your hands behind your back and look at the ceiling. Gently pull your hands towards the floor (only as far as you're comfortable).

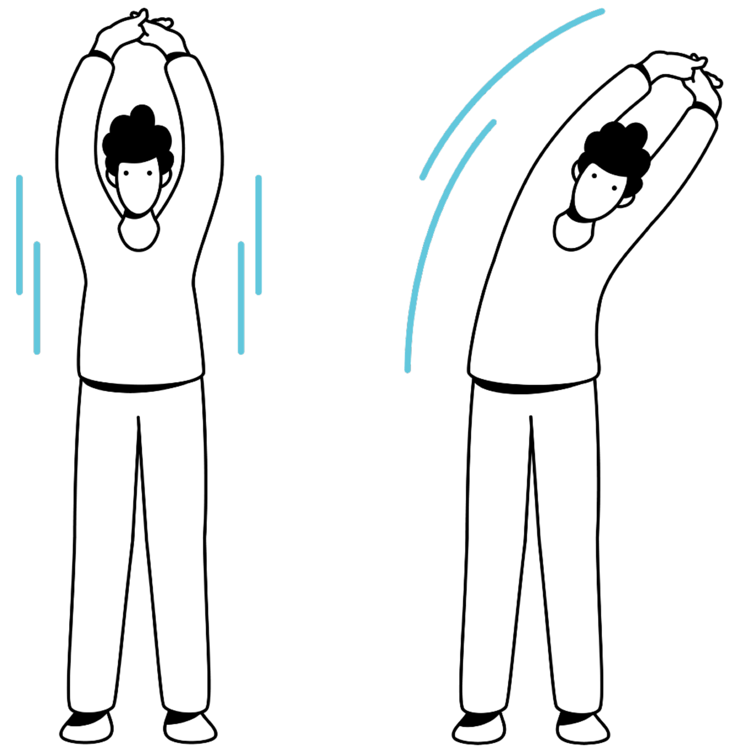

Stretch With Arms Above Head

Raise your hands above your head and stretch them from left to right. Interlink your hands when you do it for a bit more intensity.

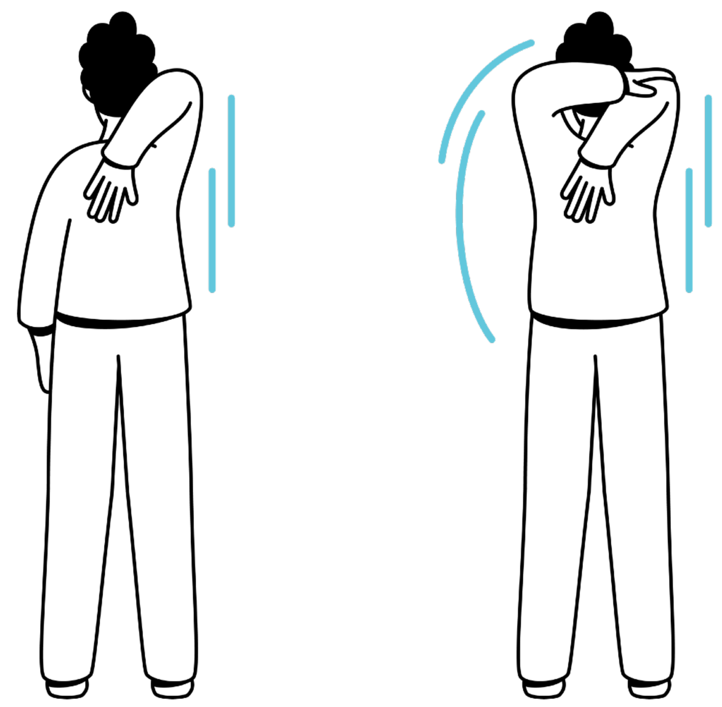

Arms Behind Back Stretch

Reach behind your shoulders and try to walk your fingers down your back. Don't worry if they don't touch - try holding on to your shirt instead.

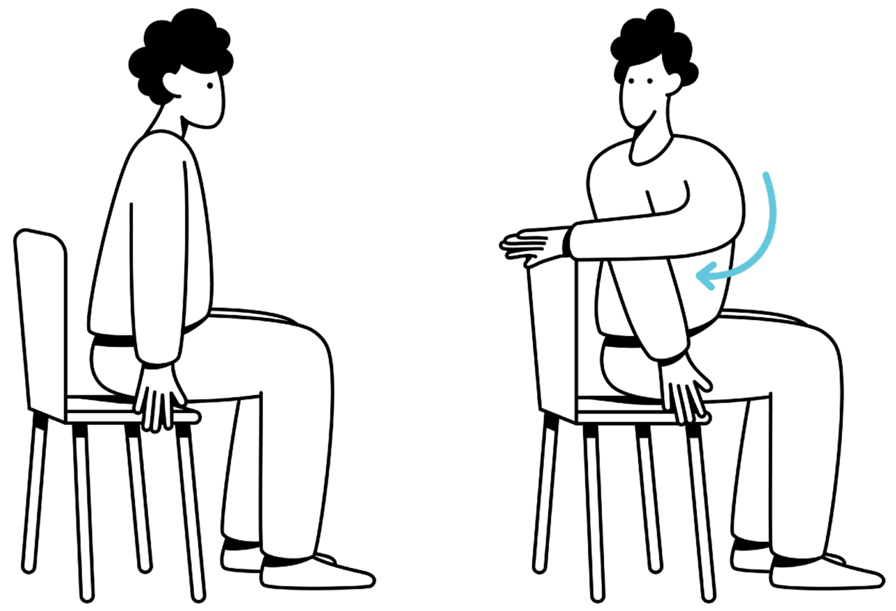

Seated Unwind

Unwind - literally! Twist from the waist, and look back over your shoulder. Hold for a few seconds before twisting the other way.

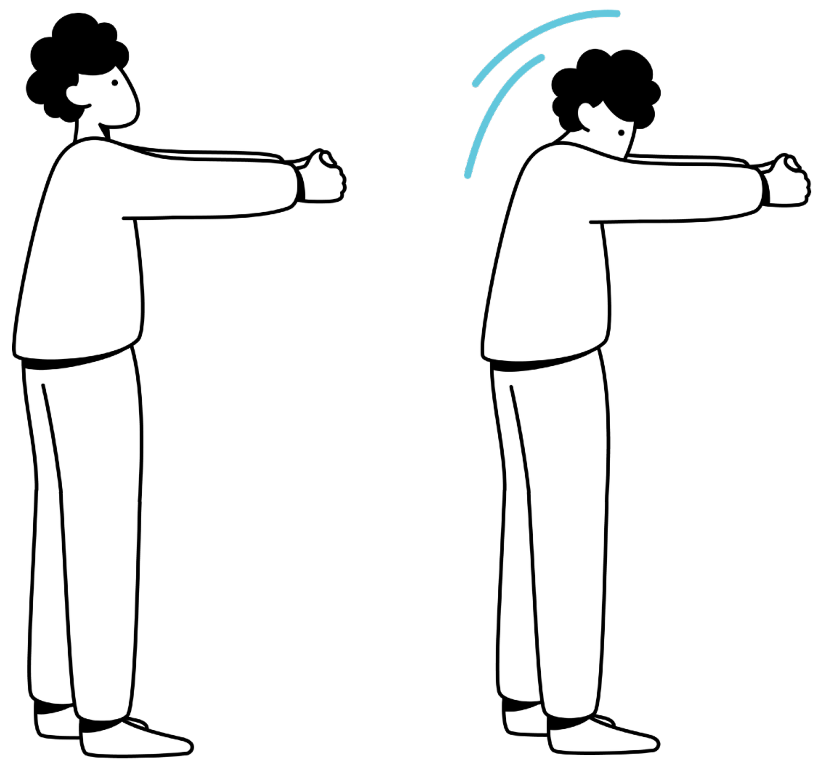

Upper Back Stretch

Keep your chin tucked into your chest
Go to where feels comfortable. Interlock hands facing you and push towards the opposite wall to stretch your shoulders and upper back.

MOOD-BOOSTING

Activities to lift your spirits and clear your head.

Doing Five in Five is simple:
Choose 5 exercises from the activity library and do each one for 1 minute, adding up to a 5 minute mini-workout.

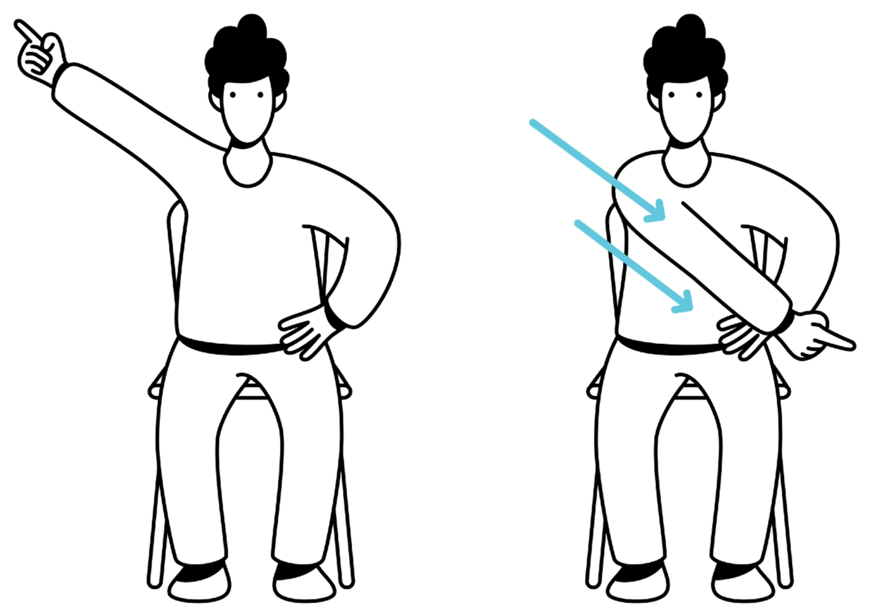

Dance In Place

Dance in place, any way that you like.
There are no wrong moves!

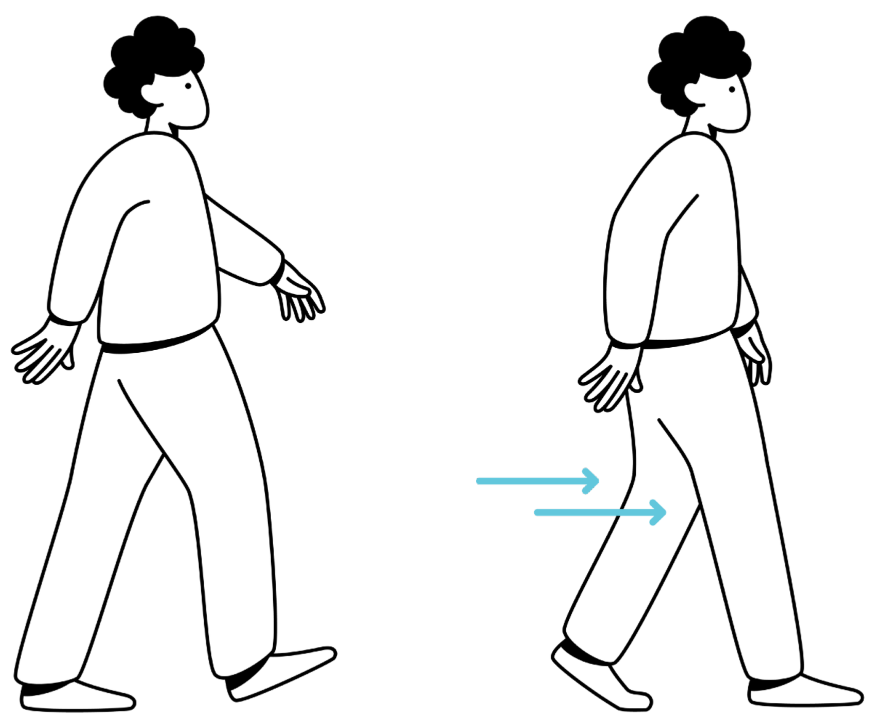

Move In Your Surroundings

Move around your surroundings for a minute, at your own pace.
If you've got some outdoor space, why not move there?

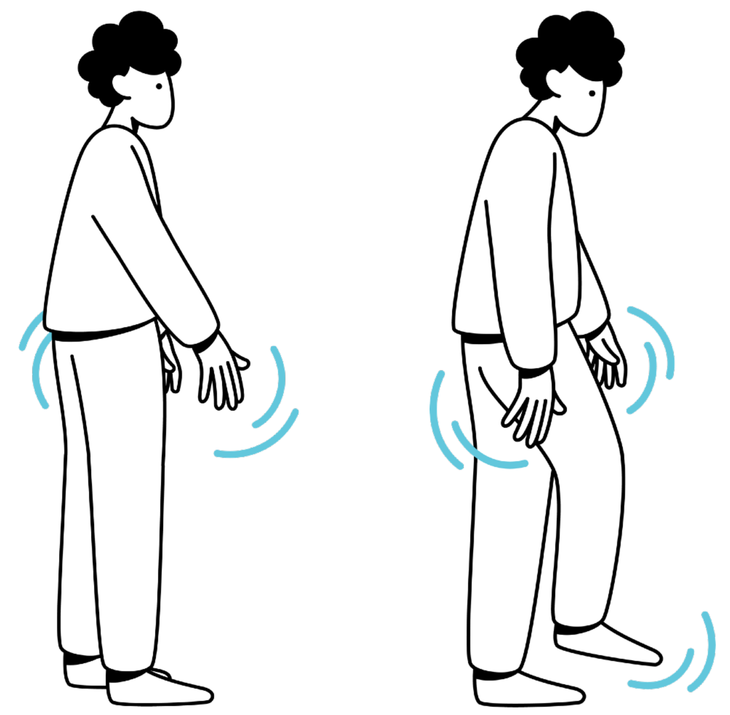

Shake It Out

Shake out the tension in your arms - and if you can, your legs, too. Now's the time to let loose!

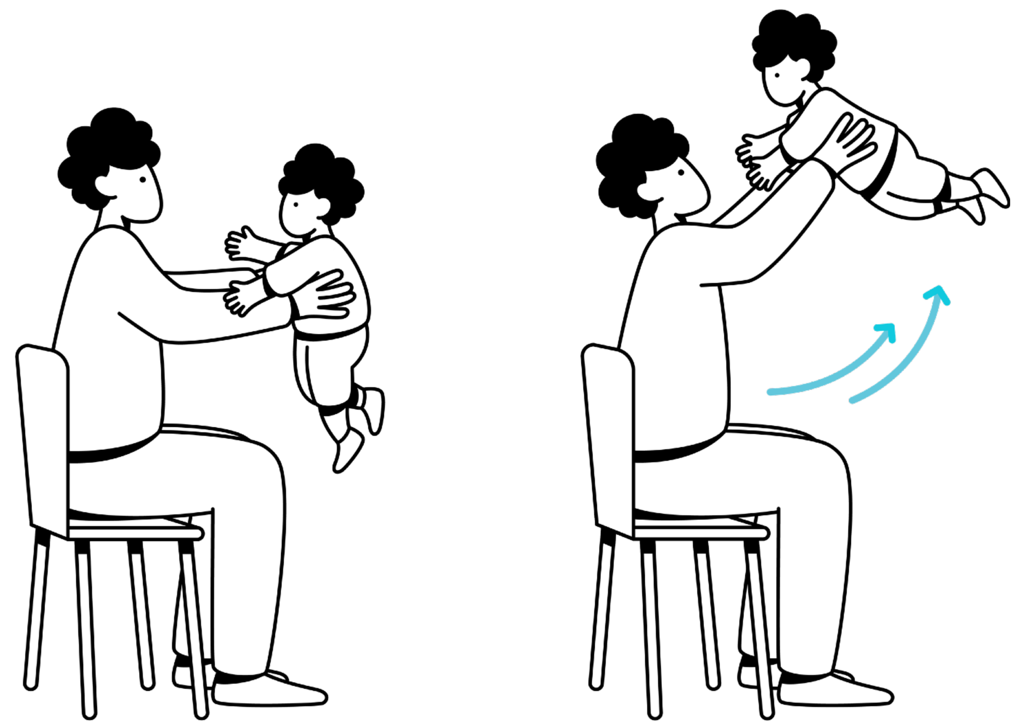

Get Active With Grandchild

If you're getting active with a child or grandchild, it's their turn to pick which move you do!

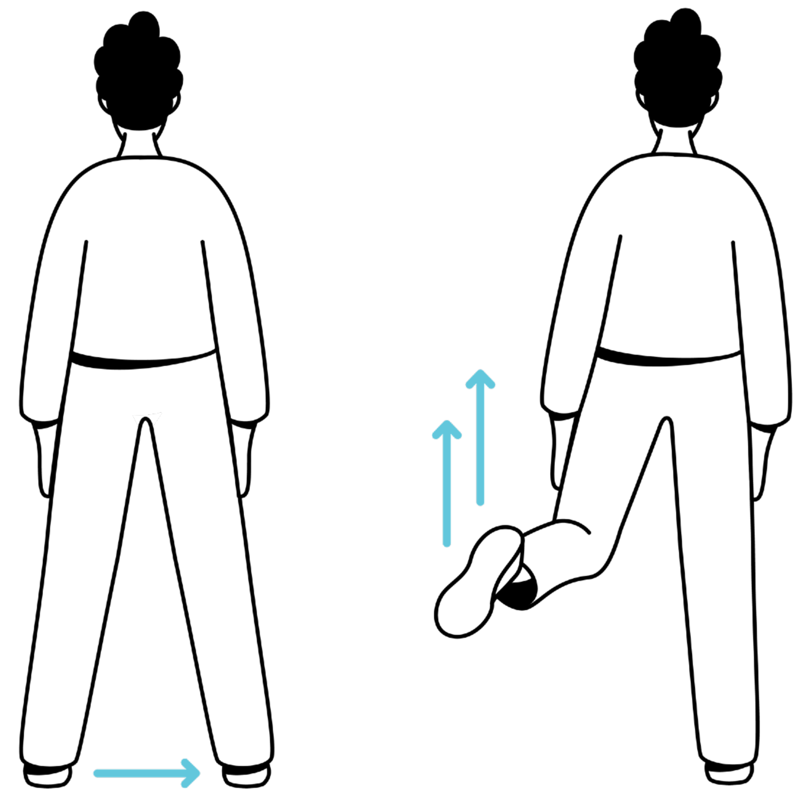

Side Step And Heel Flick

Step to the side and lift your opposite heel towards your backside. Alternate and step to the other side and repeat the movement. Add arm lift to increase intensity.

EXERCISES WITH ACCESSORIES

Exercises with equipment or household items.

Doing Five in Five is simple:
Choose 5 exercises from the activity library and do each one for 1 minute, adding up to a 5 minute mini-workout.

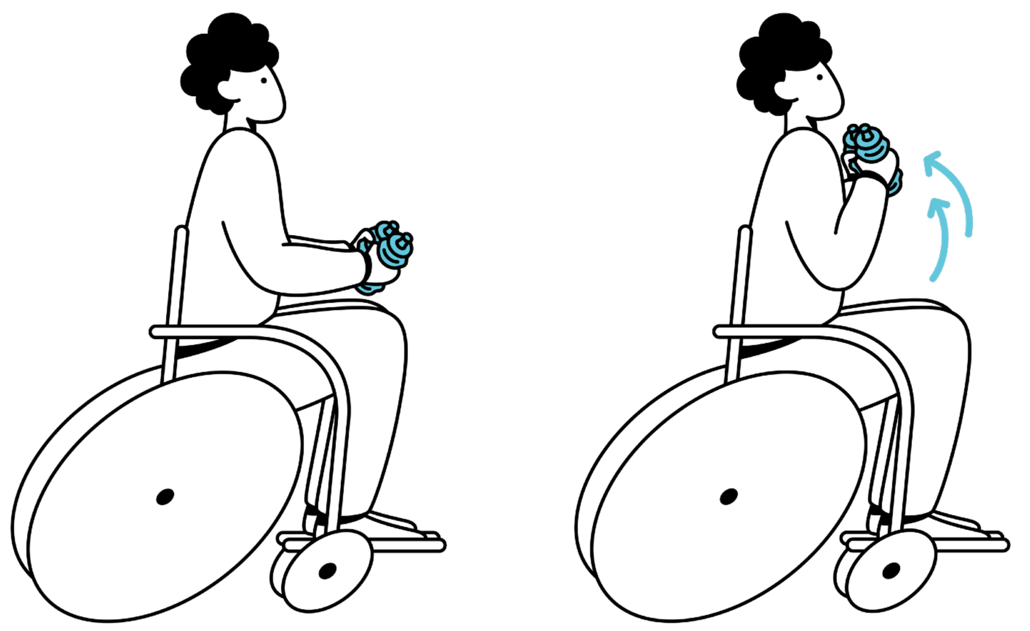

Bicep Curl With Cans

Holding a full water bottle or unopened can, curl your arm up to your shoulder and then bring it back down. No fancy equipment needed!

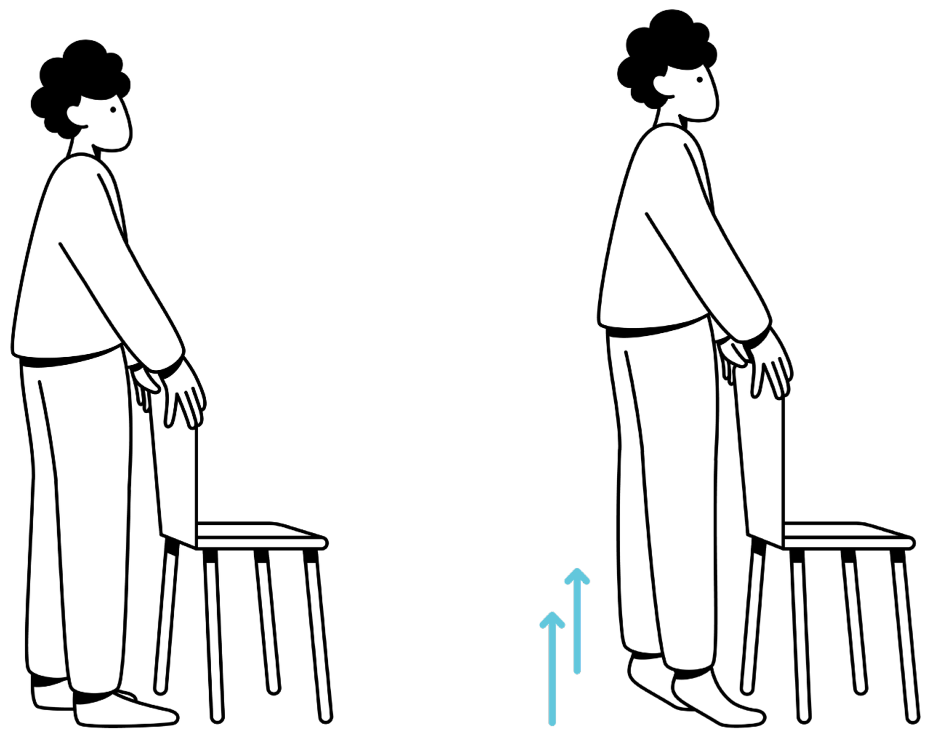

Calf Raises With Chair

Rise up to your tip toes for a few seconds before lowering back down. Hold onto a chair for support if needed.

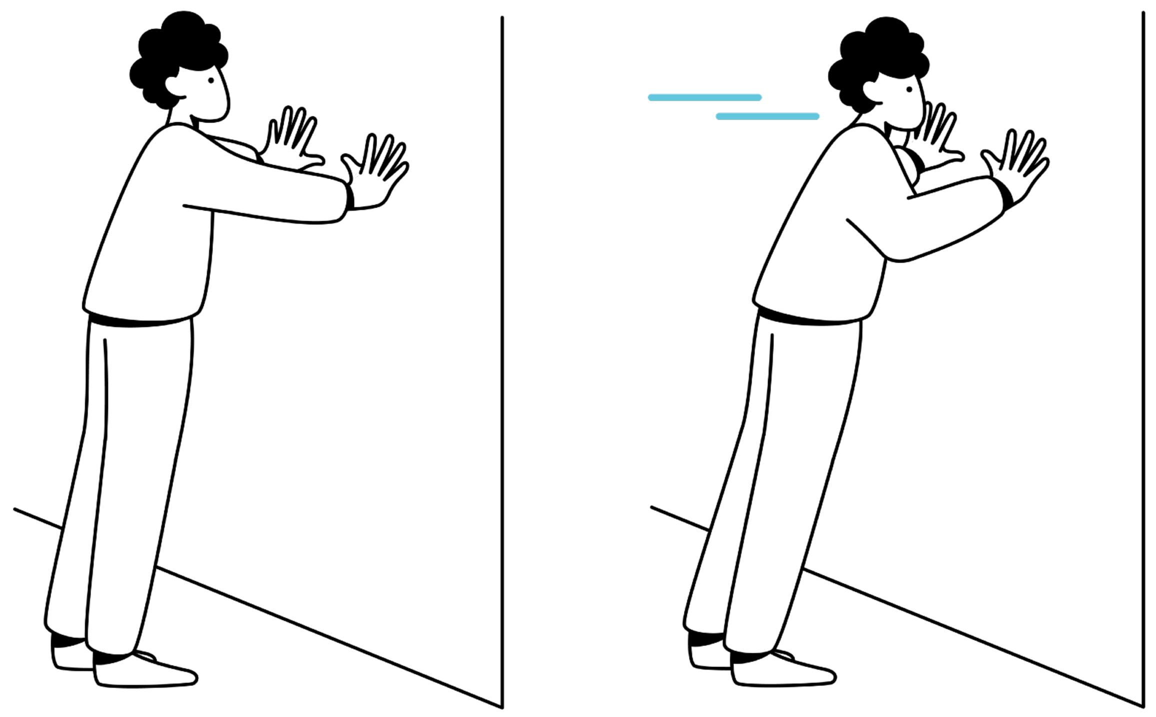

Wall Push Up

Face the wall and stand at an angle, hands shoulder width apart at shoulder height. Bend your elbows to lower your chest to the wall, then push back up.

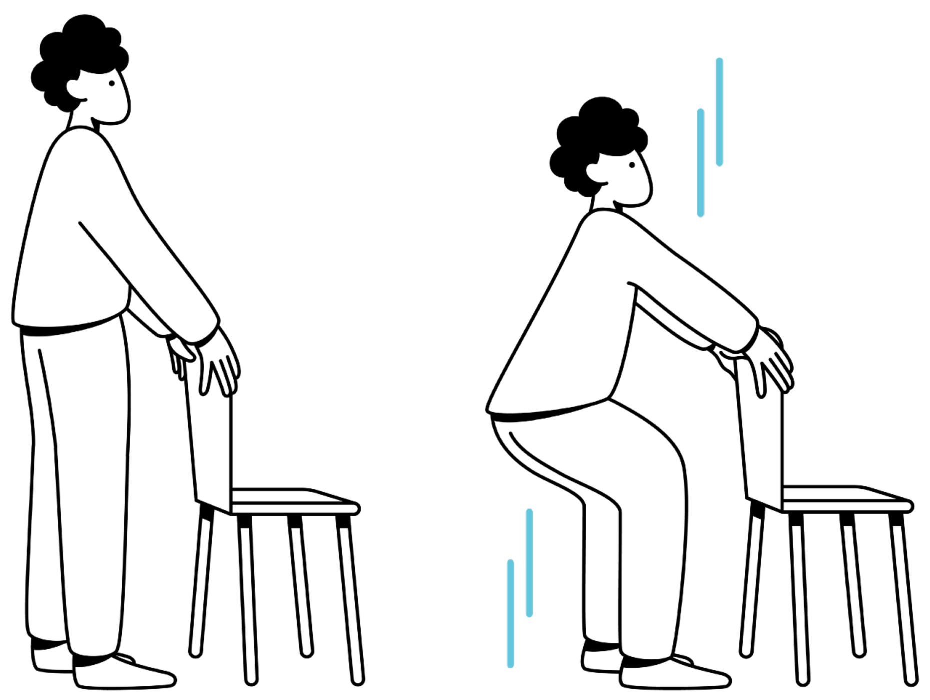

Squat With Chair

Try squatting, but with a little support. Hold onto a chair with a straight back and feet hip width apart. Bend at the knees like you're going to sit, then straighten your legs to stand right back up.

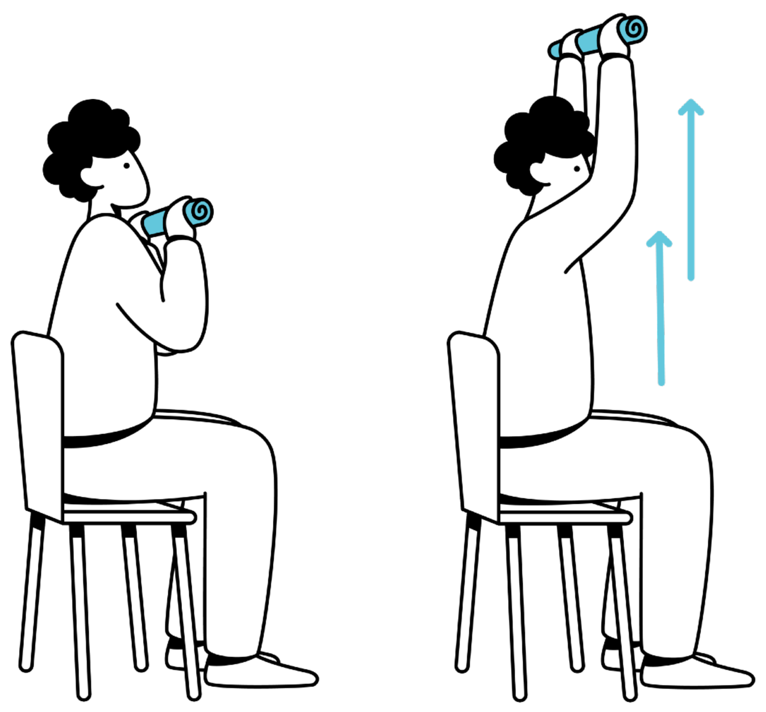

Tea Towel Resistance Band

Make your own resistance band! Pull both ends of a tea towel with your hands. Bring the towel to your chin and then lift it over your head keeping bent elbows.

WHILE WATCHING TV

Activities to get moving while the ads are on.

Doing Five in Five is simple:
Choose 5 exercises from the activity library and do each one for 1 minute, adding up to a 5 minute mini-workout.

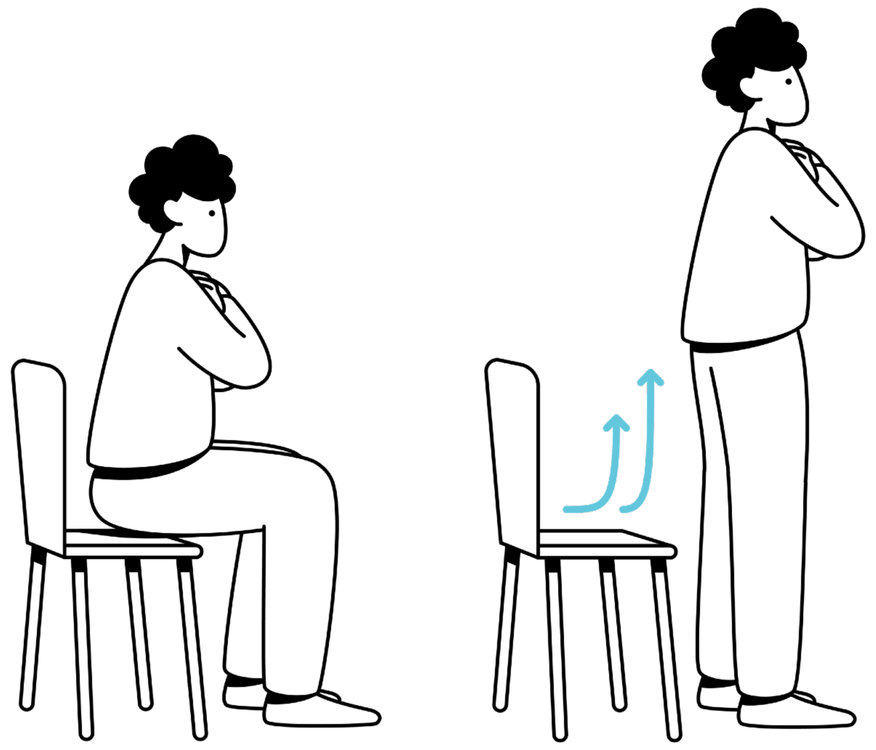

Sit To Stand

Start on the front of your chair, arms folded across your chest and stand up then slowly sit back down and repeat. Use your arms to support you if needed.

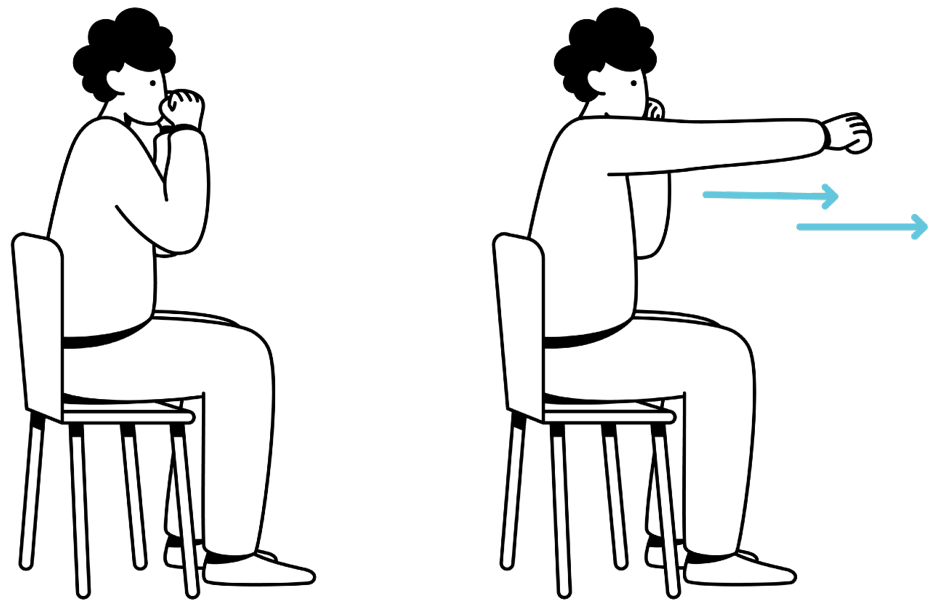

Seated Punches

While seated or standing, reach your arms out to hit an imaginary punching bag!

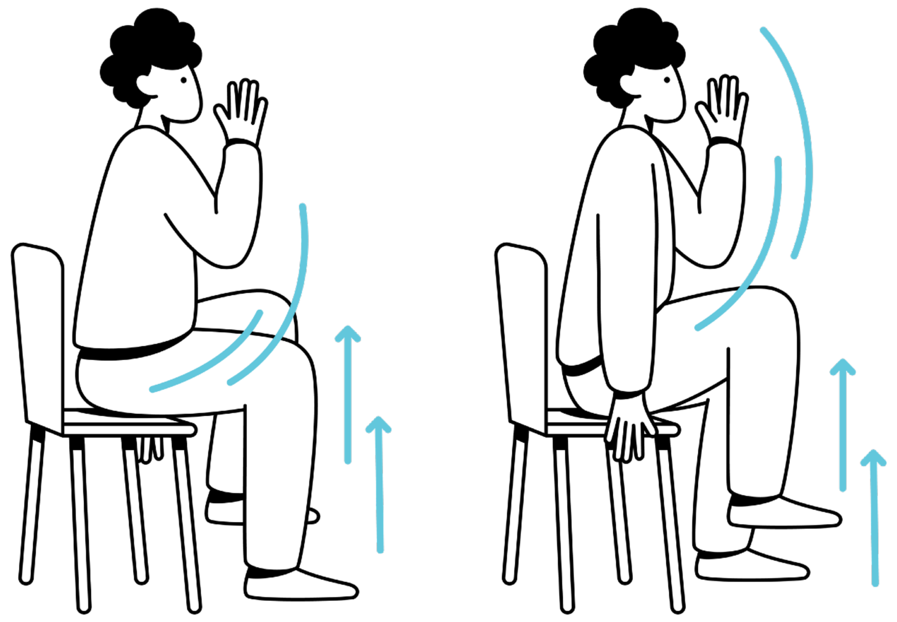

Seated March

Walk on the spot and gradually increase arm swings and knee lifts until you're marching. If you prefer to stand, you can march on the spot.

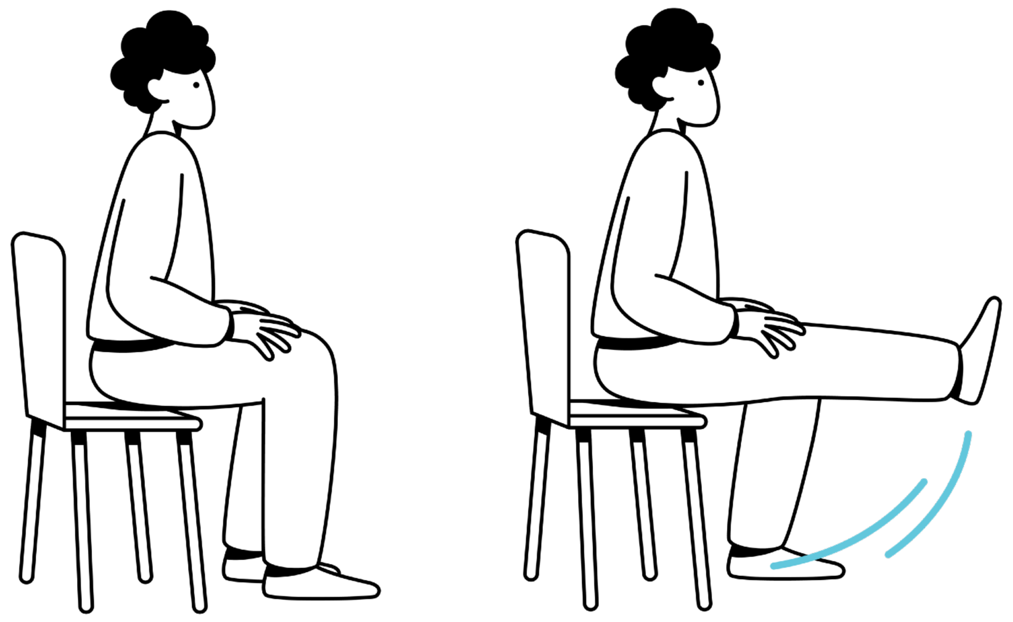

Seated Leg Extension

Sit on the front of your chair and extend the leg from the knee on alternate legs.

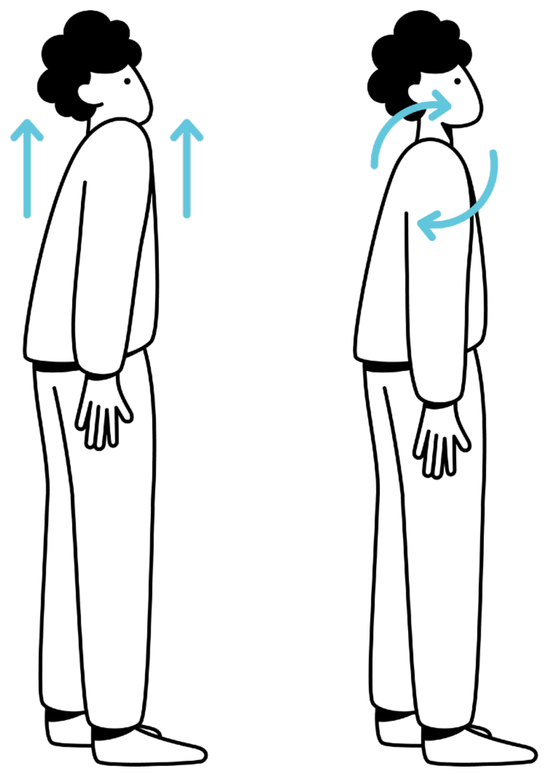

Shoulder Roll

Roll your shoulders around, first forwards, then backwards. After a little while, raise your arms for an arm roll, too!

WHILE OUTDOORS

Exercise in the garden or on the go.

Doing Five in Five is simple:
Choose 5 exercises from the activity library and do each one for 1 minute, adding up to a 5 minute mini-workout.

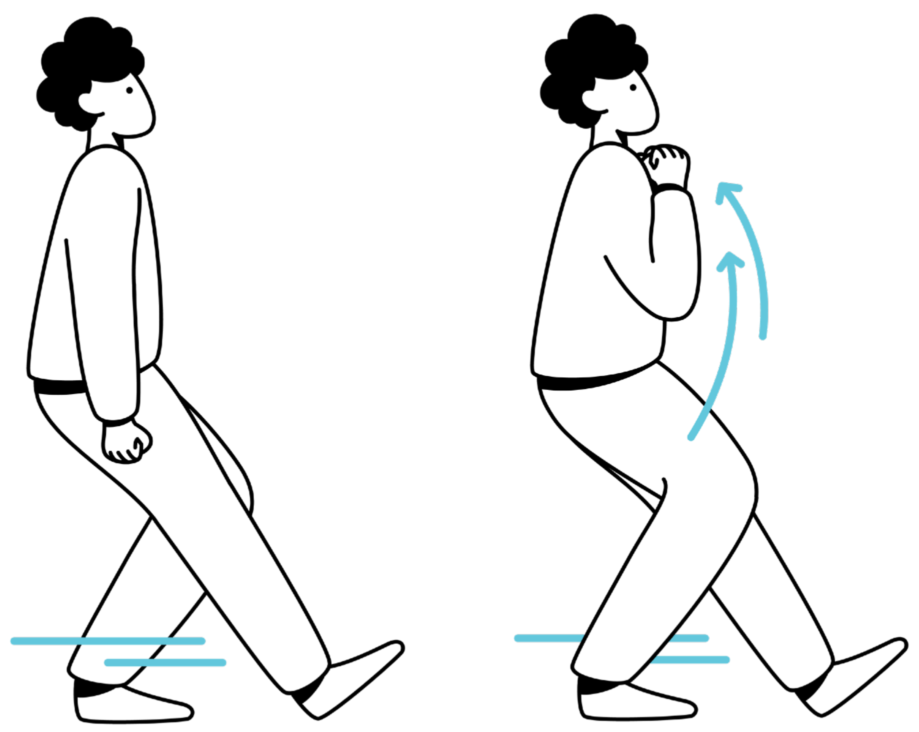

Heel Digs

Alternate your legs and place your heel in front of you with a slight bend of the opposite leg. You could add an arm curl to increase intensity.

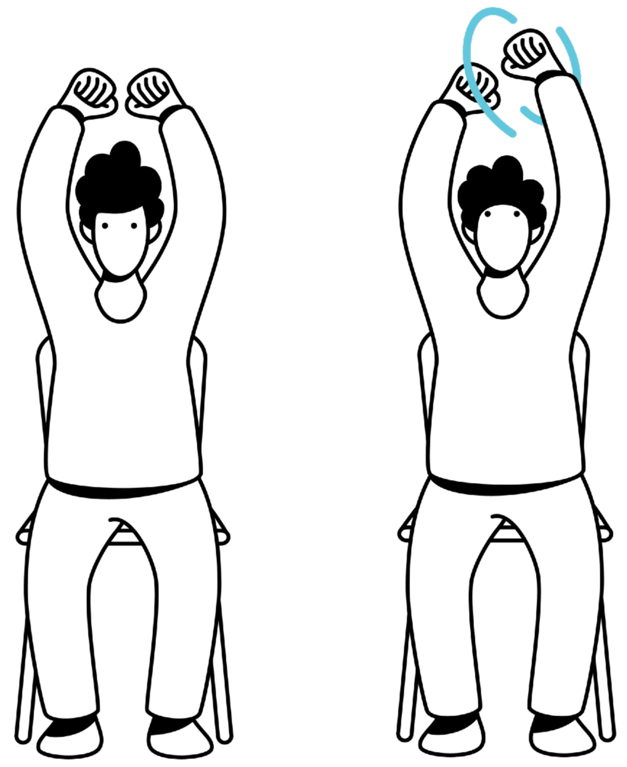

Seated Rolling Punches

Lift arms above your head, feet hip width apart, and do a rolling punching motion.

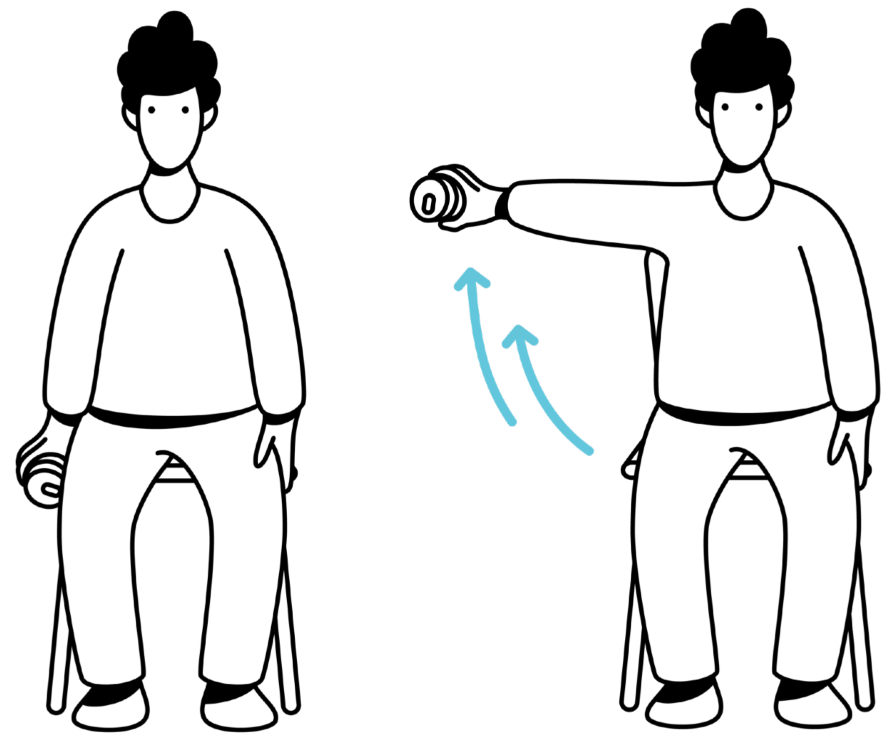

Seated Lateral Raises

Start with your arms down by your side and lift then to shoulders height laterally. Use a tin can or weight for extra intensity.

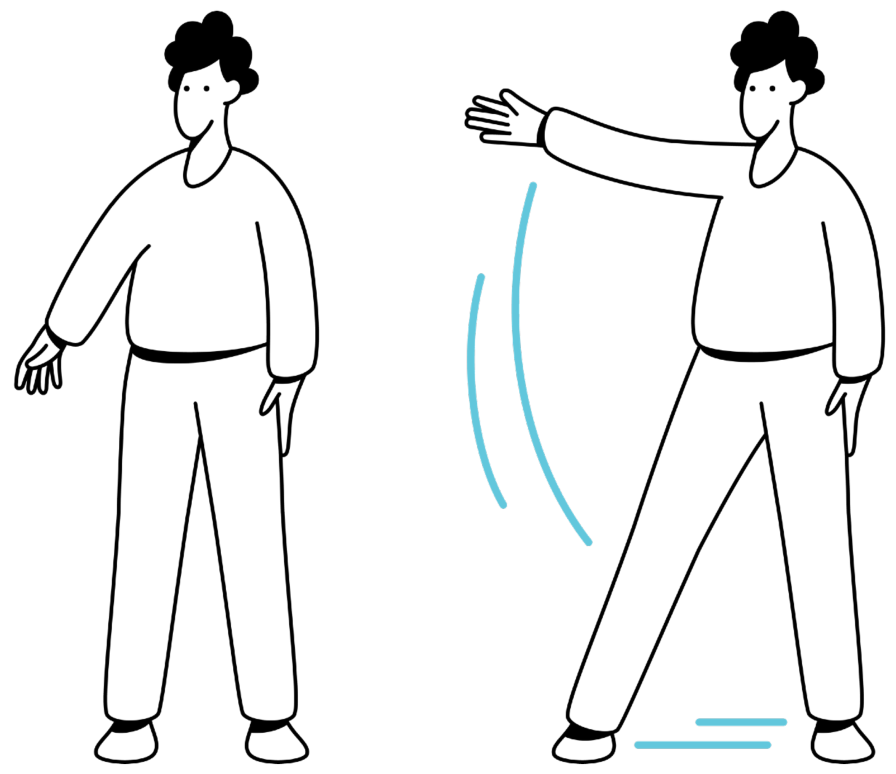

Half Jacks

Step to the side and lift one arm on your right side, then your left. If you’re seated, you can do overhead claps instead!

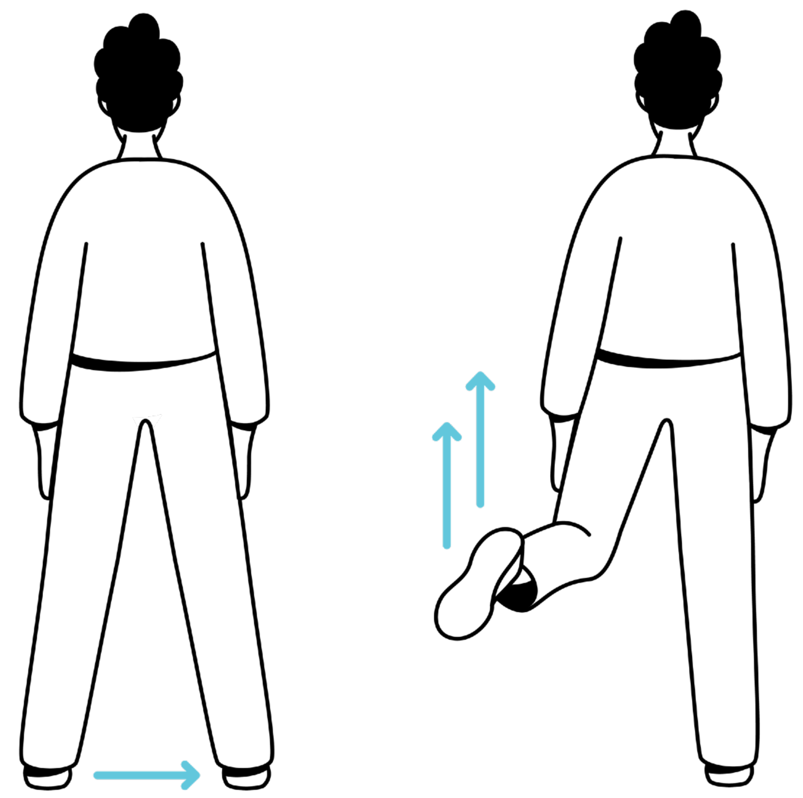

Side Step And Heel Flick

Step to the side and lift your opposite heel towards your backside. Alternate and step to the other side and repeat the movement. Add arm lift to increase intensity.

IN THE KITCHEN

Moves to do while you're making a brew.

Doing Five in Five is simple:
Choose 5 exercises from the activity library and do each one for 1 minute, adding up to a 5 minute mini-workout.

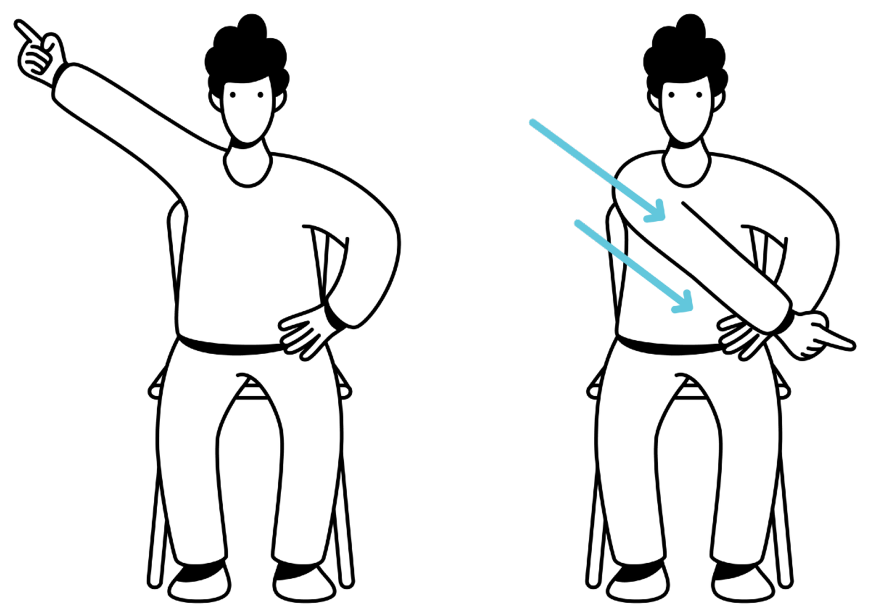

Dance In Place

Dance in place, any way that you like. There are no wrong moves!

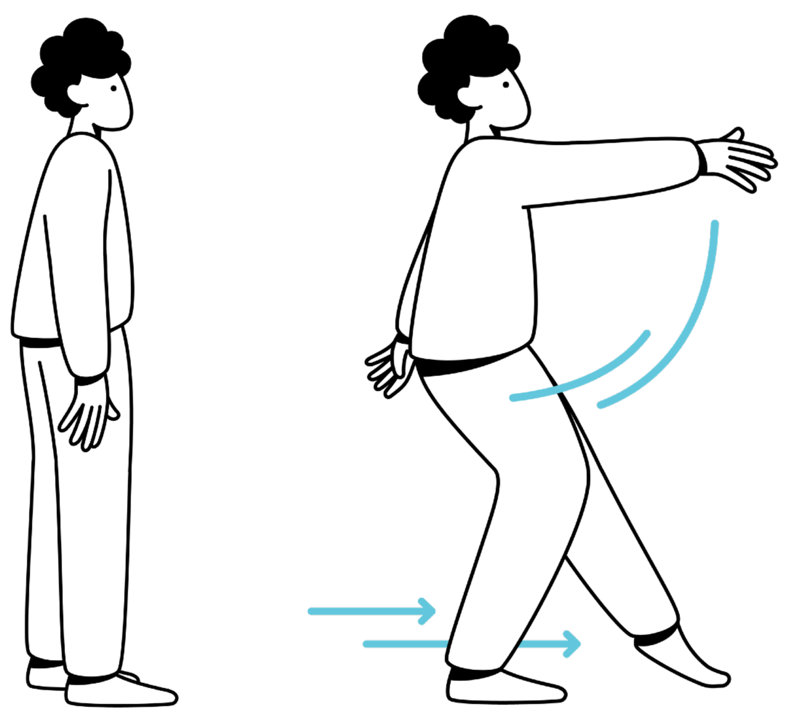

Toe Taps

Alternate your legs and place your toe out in front of you, slight bend of the opposite knee. Add arm raise, to the front, to increase intensity. Can be done seated or standing.

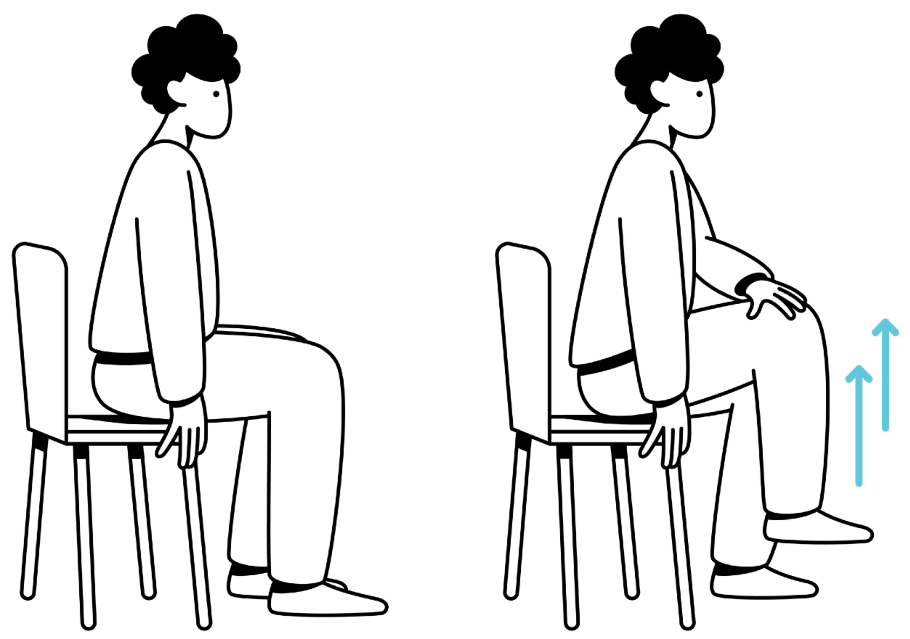

Knee Lifts

Raise knee up towards hip-height and tap your hand on to the knee when raised.

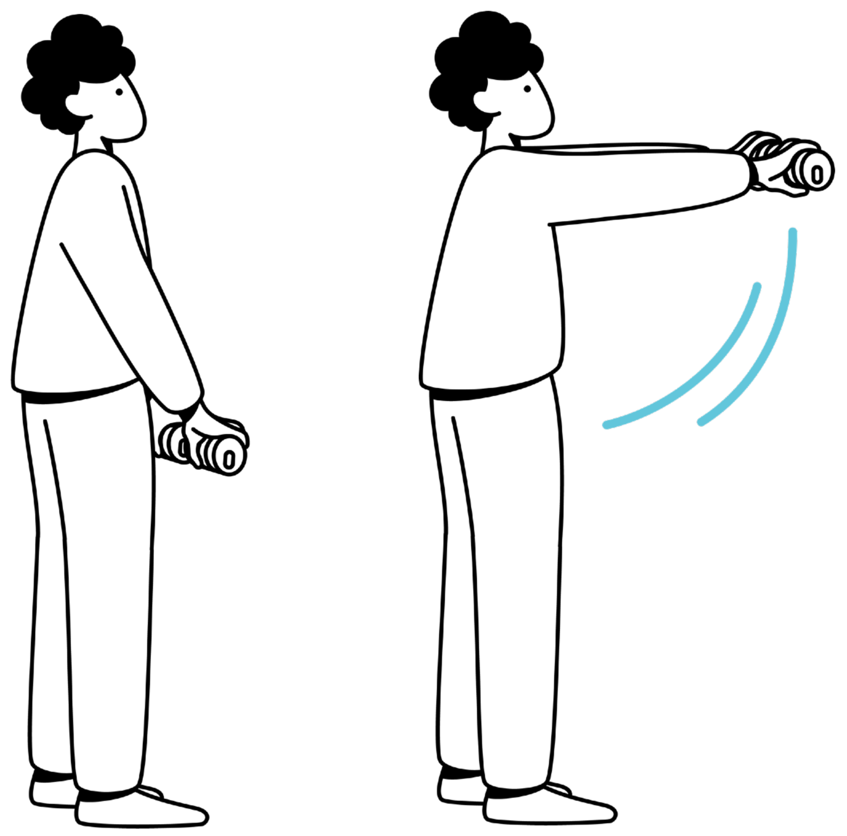

Front Raises

Grab a tin (or not) and lift your arms to the front from your side up until your arms are in line with your shoulder.

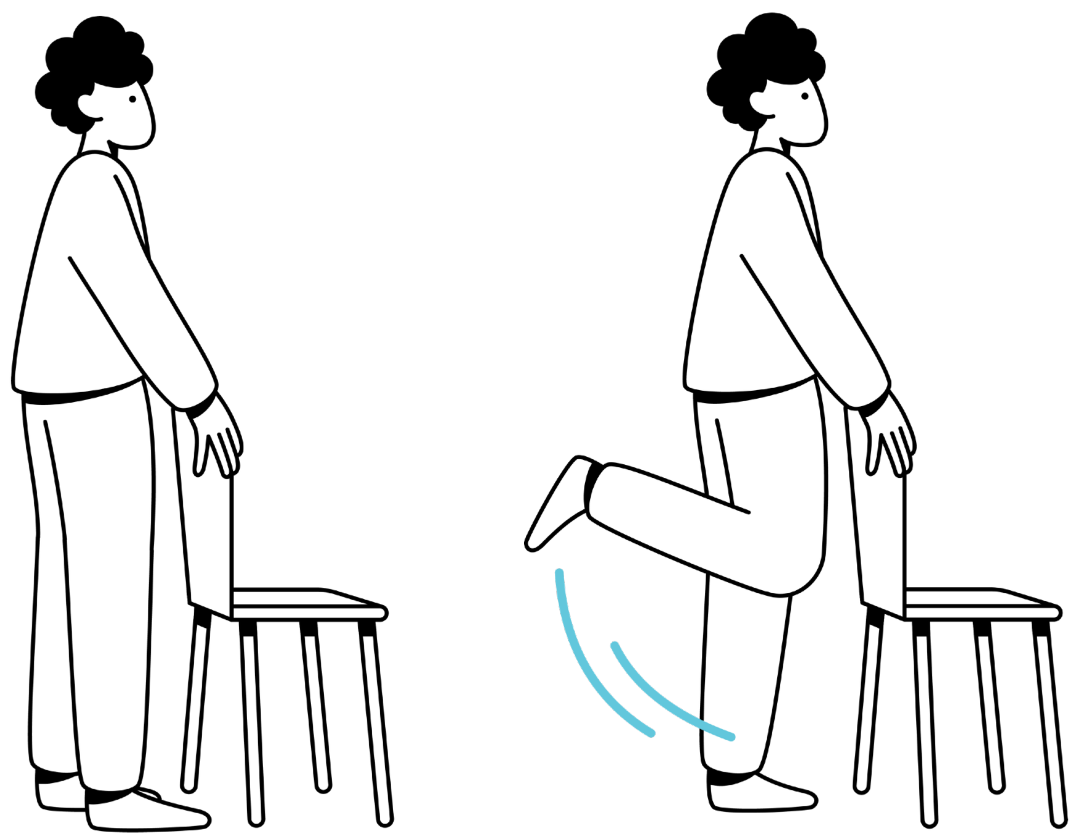

Hamstring Curl

Hold on to the worktop or a chair with one arm, stand up straight and lift one heel up at a time towards your backside.

MY FIVE IN FIVE

Pick five activities that best work for you and your health condition to build your own mini-workout plan.

1

2

3

4

5
